# Survey of ex vivo drug combination effects in chronic lymphocytic leukemia reveals synergistic drug effects and genetic dependencies

**DOI:** 10.1038/s41375-020-0846-5

**Published:** 2020-05-13

**Authors:** Marina Lukas, Britta Velten, Leopold Sellner, Katarzyna Tomska, Jennifer Hüellein, Tatjana Walther, Lena Wagner, Carolin Muley, Bian Wu, Małgorzata Oleś, Sascha Dietrich, Alexander Jethwa, Hanibal Bohnenberger, Junyan Lu, Wolfgang Huber, Thorsten Zenz

**Affiliations:** 1 Department of Molecular Therapy in Haematology and Oncology, DKFZ & NCT Heidelberg, Heidelberg, Germany; 2 Department of Internal Medicine II, Klinikum Rechts der Isar, TU München, München, Germany; 3 Genome Biology Unit, EMBL, Heidelberg, Germany; 4 Department of Internal Medicine V, Heidelberg University Hospital, Heidelberg, Germany; 5 Department of Pediatrics, University Hospital Heidelberg, Heidelberg, Germany; 6 Institute of Pathology, University Medical Center Göttingen, Göttingen, Germany; 7 Department of Medical Oncology and Haematology, University Hospital Zürich and University of Zürich, Zürich, Switzerland

**Keywords:** Preclinical research, Chronic lymphocytic leukaemia, Combination drug therapy, Translational research

## Abstract

Drug combinations that target critical pathways are a mainstay of cancer care. To improve current approaches to combination treatment of chronic lymphocytic leukemia (CLL) and gain insights into the underlying biology, we studied the effect of 352 drug combination pairs in multiple concentrations by analysing ex vivo drug response of 52 primary CLL samples, which were characterized by “omics” profiling. Known synergistic interactions were confirmed for B-cell receptor (BCR) inhibitors with Bcl-2 inhibitors and with chemotherapeutic drugs, suggesting that this approach can identify clinically useful combinations. Moreover, we uncovered synergistic interactions between BCR inhibitors and afatinib, which we attribute to BCR activation by afatinib through BLK upstream of BTK and PI3K. Combinations of multiple inhibitors of BCR components (e.g., BTK, PI3K, SYK) had effects similar to the single agents. While PI3K and BTK inhibitors produced overall similar effects in combinations with other drugs, we uncovered a larger response heterogeneity of combinations including PI3K inhibitors, predominantly in CLL with mutated IGHV, which we attribute to the target’s position within the BCR-signaling pathway. Taken together, our study shows that drug combination effects can be effectively queried in primary cancer cells, which could aid discovery, triage and clinical development of drug combinations.

## Introduction

Abnormal B-cell receptor (BCR) signaling is a key mechanism of disease development and progression in chronic lymphocytic leukemia (CLL) [1], and this is the basis for the clinical success of therapies targeting different downstream kinases. Ibrutinib, for example, is a covalent inhibitor of Bruton’s tyrosine kinase (BTK). BTK is essential for the activation of the AKT/ERK and the NF-*κ*B pathway through the BCR [2–4] and involved in B-cell adhesion and chemokine-mediated homing [5, 6]. Ibrutinib has shown activity in several hematological malignancies [7–10] and has led to high response rates in CLL [11, 12] and mantle cell lymphoma [13]. Another example is idelalisib, an inhibitor of the delta isoform of phosphatidylinositol 3-kinase (PI3Kδ). PI3Kδ is a key component of the BCR-signaling cascade [14] that mediates effects on CLL cell proliferation, migration, and survival [15]. Although inhibitors of the BCR (BCRi) have transformed the treatment of CLL, drug resistance emerges [16], and BCRi in the vast majority of cases do not lead to complete remission [17]. A promising approach to overcome such limitations are combinatorial approaches, as they may increase response rates and durabilities by circumventing potential resistance mechanisms. However, given the striking single agent activity of targeted agents (e.g., BTK inhibitors [18], PI3K inhibitors [19], and BH3 mimetics [20]) and the advent of effective combination regimens, the clinical development of new combination regimes may be held back due to a lack of opportunities for testing.

Most systematic combinatorial drug studies to date have been based on cell line models. In diffuse large B-cell lymphoma (DLBCL) cell lines, inhibitors of mTOR [21], PI3K, Bcl-2, and chemotherapeutic agents were reported to show synergistic activity when combined with ibrutinib [22]. Also, ibrutinib cooperates with lenalidomide by downregulating IRF4 [23]. While cell lines are valuable disease models, the use of primary patient material has potential advantages including the absence of subclone selection and the ability to better represent the natural molecular heterogeneity of the disease. CLL cells in the body reside in multiple compartments with different microenvironments that might alter pathway sensitivity. Here, we limit our study to primary patient material from the peripheral blood (PB), which has the advantage of being easily accessible. This implies a need for further validation of the results with respect to microenvironment-dependent modulation, but it also facilitates the application of such assays in precision medicine approaches for the donating patients [24, 25].

Hypothesis-driven studies that investigated particular combinations of drugs on primary CLL cells showed synergistic effects for the combination of the Mdm2 inhibitor nutlin-3 [26] and of the XPO1 inhibitor selinexor with ibrutinib [27]. Furthermore, ibrutinib and idelalisib combined with the Bcl-2 inhibitor venetoclax yielded synergistic effects in CLL [28, 29]. Such studies provide potential starting points for clinical development, as shown by successful trials for combination of ibrutinib and venetoclax [30, 31]. Combination of BCRi with immunotherapy has entered clinical care [32, 33], and combination of ibrutinib with chemoimmunotherapy (bendamustine/rituximab, fludarabine/cyclophosphamide/rituximab) showed the promising results in a phase II and III studies [34, 35]. A recent study combined epigenome with single-cell chemosensitivity profiling in patient samples collected before and during ibrutinib therapy and observed preferential sensitivity to proteasome, PLK1, and mTOR inhibitors during ibrutinib treatment [36]. Despite the success of some new combination regimens including ibrutinib in clinical studies [30–35], there has been a lack of larger, more systematic approaches that characterize a range of combinatorial drug treatments across representative cohorts of primary cancer cells, to search for optimal, new combinations. Here, we report an assay in which we systematically investigated drug combination effects on a set of primary CLL samples. In light of the clinical success of BCR targeting, our study focuses on combinations involving BCR inhibitors, as these are already a part of clinically relevant combination approaches in CLL and other B-cell lymphomas. We uncovered molecular subgroup-specific effects and found differences between the combinatorial effects of PI3K and BTK inhibitors specifically in CLL with mutated IGHV.

## Material and methods

### Patient samples

PB samples with high lymphocyte fractions (median 95%, ranging from 78–99%) from 52 patients of the University Hospital of Heidelberg fulfilling standard diagnostic criteria for CLL were obtained. Samples from two additional patients were used in a first experiment but not considered for the analysis due to the lack of a suitable control plate. Patients provided written informed consent in accordance with the Declaration of Helsinki and local ethical approval. Mononuclear cells were isolated by centrifugation over Ficoll-Paque Premium (GE healthcare, Freiburg) and cryopreserved.

### Cell culture and in vitro treatments

Cells were thawed in RPMI-1640 supplemented with 5% FBS, 1% penicillin/streptomycin, and 1% glutamine. We used 40,000 primary cells per well, cultured in 50 µl RPMI supplemented with 10% pooled and heat inactivated AB-type human serum (RPMI-HS, Biomedicals) at 37 °C, 5% CO_2_. The drugs and cells were dispensed using an electronic pipette (Millipore) into 384 flat bottom Greiner plates. The plates were covered with breathe easy sheets and Greiner cell culture lids to prevent evaporation and incubated at 37 °C with 5% CO_2_ for 48 hours.

### Compounds and combinatorial drug screen

**Fig. 1:**
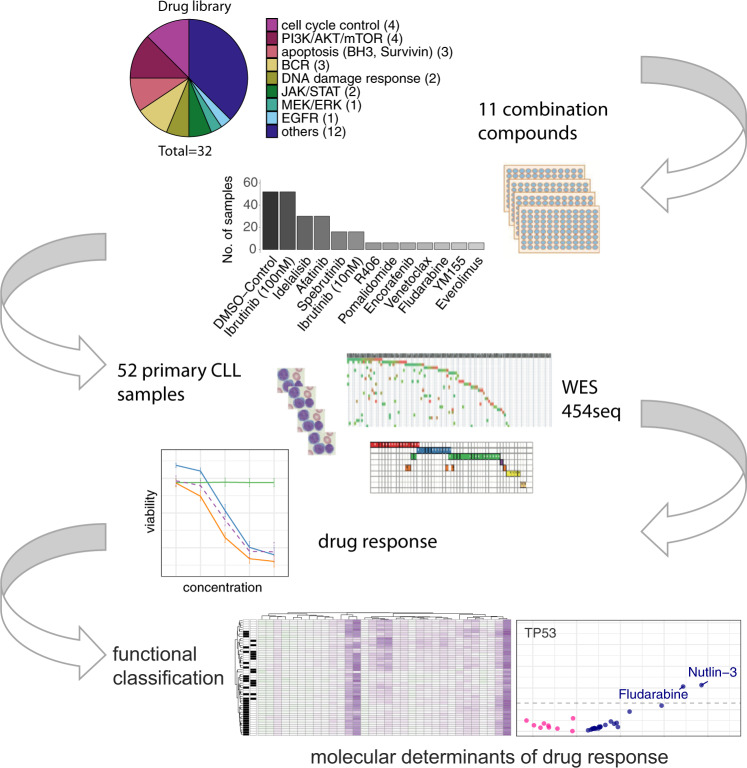
Systematic identification of rational drug combinations in CLL. Study outline summarizing ex vivo drug response assessment of 52 primary CLL samples. A combinatorial screen pairing 11 combination drugs at 1–2 concentrations with 32 library drugs at 5 concentrations inhibiting key oncogenic pathways was established. Each experiment included combinations of the library drugs with a control (DMSO), ibrutinib 100 nM and two additional combination drugs. Viability was assessed after 48 h based on ATP luminescence. Interesting drug combinations were identified using an independent effect model and the Highest Single Agent approach (“Material and methods”). Molecular determinants of drug response were analyzed based on genomic characterization by whole exome sequencing, targeted sequencing and IGHV analysis.

Drugs (*n* = 34, Supplementary Table 1) were obtained from commercial suppliers and kept at a concentration of 10 mM in DMSO. Each of a set of 32 drugs (termed library drugs in the following) was combined with each of a set of 11 drugs (termed combination drugs) (Fig. 1); two combination drugs were not part of the library, thus a total of 34 drugs were used. The drugs were chosen from clinically used drugs (including fludarabine, ibrutinib, venetoclax) and other inhibitors of pathways of importance in CLL. We prepared a master plate by diluting each library drug using the same doses as described by Dietrich et al. [37]. In short, all 32 library drugs were tested at 5 concentrations, i.e., with fourfold serial dilutions starting at 1 µM for navitoclax, venetoclax, SNS-032, YM155, and doxorubicine, 4 µM for arsentrioxide, 7 µM for chaetoglobosin A and 10 µM for all other drugs. For each experiment, drugs from the master plate were diluted with 10% pooled and heat-inactivated AB-type human serum (RPMI-HS) and transferred to 384-well plates (Greiner) using electronic pipettes (Millipore). We then added to these wells each of 11 combination drugs at 1 or 2 concentrations (100 nM for ibrutinib, idelalisib, spebrutinib, R406, afatinib, fludarabine, pomalidomide, everolimus, and encorafenib; 10 nM for YM155 and ibrutinib, and 5 nM for venetoclax) and tested each combination on the samples from 6 to 52 patients. The highest number of CLL samples were analyzed for the combination drugs ibrutinib (*n* = 52 patient samples), idelalisib (*n* = 30), afatinib (*n* = 30), and spebrutinib (*n* = 16) (Fig. 1). Based on similarity of observed responses for combinations with ibrutinib 10 and 100 nM in the first 16 patient samples, only combination with ibrutinib 100 nM was tested in the further 36 patient samples (Supplementary Fig. 1).

### Validation experiments

Measurements of 10 × 10 concentrations were assayed with selected patient samples. Navitoclax, venetoclax, and afatinib were tested in combination with ibrutinib with twofold serial dilutions starting at 2 µM (two patient samples) and 0.2 µM (three samples) for navitoclax, at 0.1 µM for venetoclax, at 5 µM for afatinib (three samples), and at 20 µM for afatinib (two samples) and ibrutinib.

### Cytotoxicity assay

To assess cell viability in the primary screen and in the validation experiments, the cells were incubated with the drugs for 48 h (based on the results of Dietrich et al. [37]), and then 12 µl of CellTiter Glo luminescent cell viability assay reagent (Promega) was added using an electronic pipette (Millipore). Plates were incubated for 15 min at room temperature. Luminescence was measured using a microplate reader (Tecan) with an integration time of 200 ms.

### Molecular characterization

To explore molecular underpinnings of drug response heterogeneity, we used whole exome sequencing for paired tumor and normal samples (*n* = 52), targeted 454 sequencing (*n* = 52), assessment of IGHV status (*n* = 52), assessment of structural variants (*n* = 52), genome-wide DNA methylation profiles (450k microarrays) (*n* = 52), and RNA sequencing (*n* = 52), as previously reported [37]. Supplementary Fig. 2 gives an overview of the patients’ somatic mutation landscape. IGHV mutation status was determined as described by Ghia et al. [38]. The variant allele frequency (VAF) of mutations was determined, but not included in the analysis. The minimum VAF of the *TP53* mutant samples in our study was 5% (see Supplementary Fig. 3).

### PamGene tyrosine kinase array

Kinase activities of primary CLL samples were measured using the PamStation® 12 system in combination with PamChip® Tyrosine Kinase Array Chips according to the manufacturer’s instructions (www.pamgene.com). Briefly, primary CLL cells were lysed in M-PER Mammalian Extraction Buffer (Pierce). Cells were treated with 1.67 µM afatinib before or after cell lysis. 2 μg of cleared cellular lysate was mixed with 4 μl 10× protein tyrosine kinase reaction buffer (PK), 0.4 μl 1 M DTT, 0.4 μl 100× BSA, 1 μl 4 mM ATP, and 0.3 μl 1 mg/ml monoclonal antiphosphotyrosine FITC conjugate (clone PY20) adjusted to 40 μl with distilled H_2_O. All chemicals were provided by PamGene International BV. Each array was blocked with 0.2% BSA and washed with PK solution. Afterwards, kinase reaction was carried out at 30 °C. The reaction mix was pulsed back and forth through the porous material of the PamChip. Every fifth cycle a picture with a built-in CCD camera and after 60 cycles five additional pictures with increasing exposure time were taken.

Spot intensities were normalized to local background signal by subtracting the median background signal from the median spot intensity, using the BioNavigator (PamGene) software. To increase the dynamic range of the measurements, the five pictures taken after 60 cycles were summarized into a single value by determining the slope of these signals as a function of exposure time, scaled by a factor of 100. The resulting values of the three biological replicates were logarithm transformed and averaged. The upstream tyrosine kinase responsible for on-chip peptide phosphorylation was predicted by using the “PamApp for PTK upstream Kinase Analysis” within the BioNavigator software (PamGene). This analysis is based on documented kinase-substrate relationships and in silico predictions from the phosphoNET database.

### Statistical analysis

The luminescence values per well were divided by the median of the DMSO-controls of each plate, resulting in values that indicate relative viability compared with the control; in the following, we term these viability values. As multiple wells on each plate contained the combination drug as a single agent we used the median of their viability values as a measure of the single agent effect for the plated sample. For the library drugs, every plate contained one well per concentration, which provided a viability value for the effect in combination or as a single agent in the plated sample. For four patient samples, we acquired replicate measurements; these were highly similar (Supplementary Fig. 4) and the average values were used for subsequent analyses. Measurements of viability values above 1.4 (corresponding to the 99.9% quantile of the data) were excluded from the analysis (i.e., treated as missing values), as these are likely outliers due to technical reasons. To map the landscape of combinatorial effects of drugs and to identify synergistic combinations, the effects of combinations were assessed by comparison to an independent effect model, as follows. For each combination of two drugs *A* and *B*, we compared the measured viability *v*_*AB*_ for the combination to the expected viability value given by the product of the corresponding single drug effects $v_{AB}^ \ast = v_Av_B$. We defined a synergy index (SI) as the average of the difference, $v_{AB}^ \ast \left( c \right) - v_{AB}\left( c \right)$, taken across the five concentrations *c* of the library drug for each patient sample. In particular, viability measured lower than predicted resulted in SI > 0, indicating synergy. To summarize drug combinations across patient samples, the median SI was used. This approach to assess synergistic effects is strongly related to the Bliss-independence model [39–42], and it shares its basic assumption of effect independency. In contrast to the Bliss model, we also considered combination effects with drugs that had a prosurvival effect, i.e., we included viability values above 100%. While the Bliss Index is often defined as the quotient of the expected and measured combination effect, here we used instead their difference to make the SI more numerically stable for small values of *v*_*AB*_ (i.e., drug combinations with strong effects). To test significance of synergistic combinations we used a one-sided paired *t*-test on the viabilities from the independence model and the measured viability from the drug combination across patient samples, separately at each concentration.

In addition, drug combination effects were also quantified with the highest single agent (HSA) approach [40, 43]. Here, we thresholded all data to lie between 0 and 100% and defined the effect of a drug A based on the viability values *v*_*A*_ as $e_A = 1 - {\mathrm{min}}\left( {1,v_A} \right)$. The HSA combination index is then given by:

$${\mathrm{CI}_{\mathrm{HSA}}} = e_{AB} - {\mathrm{max}}\left( {e_A,e_B} \right),$$

with a value greater than 0 indicating that the combination has a stronger effect than each single agent on its own. To test significance of synergistic combinations we used a one-sided paired *t*-test of the maximal single effect and the combined effect at each concentration across patient samples.

The *p* values resulting from the *t*-tests were adjusted for multiple testing across all drug–drug combinations and concentrations (*m* = 1920 tests) using the Benjamini–Hochberg procedure [44], separately for the two types of combination index (i.e., SI and HSA).

Both approaches above are based solely on drug effects at single concentrations and, whilst providing useful screening criteria for potentially interesting combinations, they might also identify less interesting cases. In particular, strongly nonlinear single agent dose–response relationships in the considered concentration range (such as for example observed with YM155, afatinib, venetoclax, and fludarabine) can result in high values of SI and/or CI for noninteresting combinations, including combination of the drug with itself. To account for this limitation and to complement the effect-based combination scores at single concentrations we used the 10 × 10 validation experiments to assess the synergy for individual patient samples using the ZIP score [45] (as implemented in the R package synergyfinder), which compares the potency of the dose–response curves between single drugs and their combination.

To assess associations of genetic features (e.g., IGHV status and *TP53* mutation) with drug responses, Student’s *t* test with equal variance between groups was performed between the corresponding groups for each single drug or drug combination that had at least three samples in each group (i.e., M-CLL/U-CLL or *TP53*wt/mut). Resulting *p* values were adjusted for multiple testing using the Benjamini–Hochberg procedure [44] across all tested genetic features, tested set of drugs (i.e., all drug combinations or all library compounds) and concentrations leading to a total of *m* = 2226 or *m* = 320 tests.

## Results

### Landscape of drug response and modulation by genetic features

**Fig. 2:**
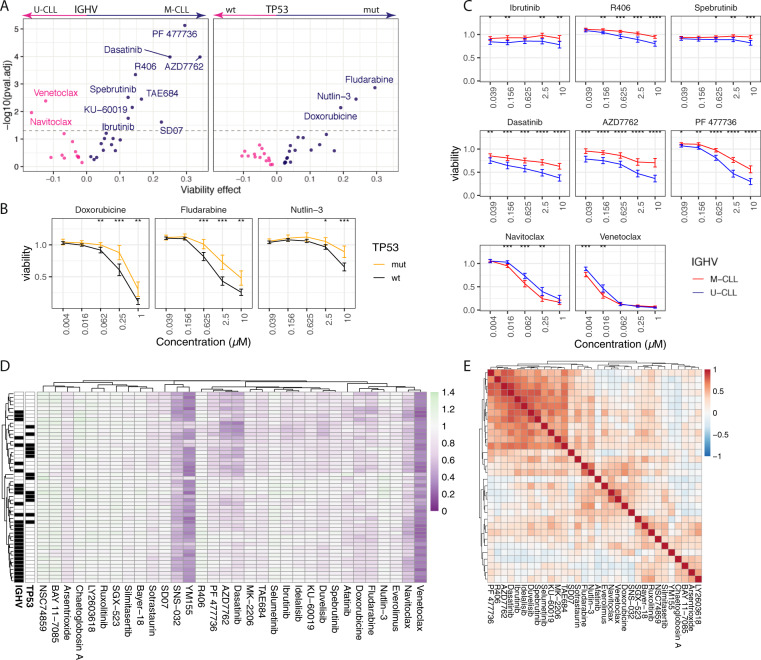
Ex vivo drug effects in CLL. **a** Volcano plot showing associations of drug responses with IGHV (left) and TP53 (right). For each drug, the five concentrations were tested separately and the most significant concentration is shown. The *x*-axis shows the mean difference in viability. Positive difference indicates higher sensitivity of U-CLL/TP53 wild type compared with M-CLL/TP53 mutated. The *y*-axis shows the logarithms of *p* values obtained from a Student’s *t* test and adjusted for multiple testing across both settings and all tested drugs and concentrations. The dashed line indicates a FDR-threshold of 5%. Significant differences for IGHV were evident for combinations with BCR-pathway inhibitors (Spebrutinib, R406), CHK inhibitors (PF477736, AZD7762), and dasatinib. Significant differences for TP53 were revealed for fludarabine and nutlin-3. **b** Drug response curves of p53-interfering drugs fludarabine, nutlin-3, and doxorubicine. Patient samples are grouped according to TP53 mutational status and shown is the mean viability within each group. Error bars denote two standard errors. The drugs are significantly more active in TP53 wild-type samples at multiple concentrations. *p < = 0.05, **p < = 0.01, ***p < = 0.001,****p < = 0.0001. **c** Drug response curves as in (**b**) for selected drugs stratified by IGHV mutational status. The three BCR inhibitors, dasatinib, and two CHK inhibitors in the upper and middle rows are significantly more active in U-CLL samples at multiple concentrations. For the two Bcl-2 inhibitors venetoclax and navitoclax higher sensitivity is observed in M-CLL samples. **d** Heatmap showing the response to the library drugs (columns) across all patient samples (*n* = 52, rows). Color code indicates viability values. Samples are annotated by IGHV and TP53 mutational status (black = mut/M-CLL, white = wt/U-CLL). Unsupervised clustering identified a BCR/CHK inhibitor-based gradient, which separates M- and U-CLL. **e** Pearson correlations for each pair of drug responses across all 52 CLL patient samples are shown. Each matrix element corresponds to a correlation coefficient, red indicating positive and blue negative values. Based on the clustering, patterns of high correlation emerged for drugs targeting the same pathways (e.g., Bcl-2 inhibitors and BCRi/CHK inhibitors).

First, we evaluated the effect of 32 library drugs applied as single agents in 52 primary CLL samples ex vivo. For this, we created dose–response curves based on 5 concentrations using ATP-based viability assessment 48 h after drug exposure, resulting in 8320 (32 × 5 × 52) measurements (“Material and methods”). To explore associations of recurrent genetic features (TP53 mutations and IGHV status) with drug sensitivities, we tested for differential drug responses between the sample groups with and without each of these mutations (*t*-test, FDR < 5%, Fig. 2a). This analysis confirmed previously reported associations, including increased sensitivity of *TP53* wild-type samples to fludarabine, doxorubicin, and nutlin-3 (Fig. 2b) and increased sensitivity of CLL samples with unmutated IGHV status (U-CLL) to BCRi, e.g., spebrutinib, R406, and ibrutinib, as well as two checkpoint kinase (CHEK) inhibitors (Fig. 2c) that we recently reported to target the BCR [37]. In addition, we tested for associations of drug responses with the DNA methylation status of the samples according to Oakes et al. [46]. We found that a low-programmed DNA methylation status of the samples was associated with increased sensitivity toward inhibitors of BCR and BCR downstream targets as well as of CHK, a finding consistent with earlier studies [37] (FDR < 5%, Supplementary Fig. 5), while the high-programmed methylation cluster displayed increased sensitivity toward Bcl-2 inhibitors and doxorubicin. These findings were also recovered by unsupervised clustering of samples by their response profiles to the 32 agents across concentrations: we observed a kinase inhibitor-based gradient with increased sensitivity toward kinase inhibition for samples that were largely U-CLL (Fig. 2d). Conversely, we considered the (dis)similarities of the drugs by computing for each pair of drugs the correlation coefficient across the 52 CLL samples. Drugs with similar functional mechanisms clustered together with a dominant cluster of kinase inhibitors targeting BCR, the downstream targets MEK or AKT or interfering with the BCR-signalosome (e.g., AZD7762) (Fig. 2e). Likewise, navitoclax and venetoclax, both targeting Bcl-2, or fludarabine and nutlin-3, both TP53 dependent drugs, clustered together. Altogether, these findings indicate that our assay platform produces biologically relevant data that are consistent with the previous results and known drug mechanisms.

### Combinatorial drug screen on primary CLL cells

**Fig. 3:**
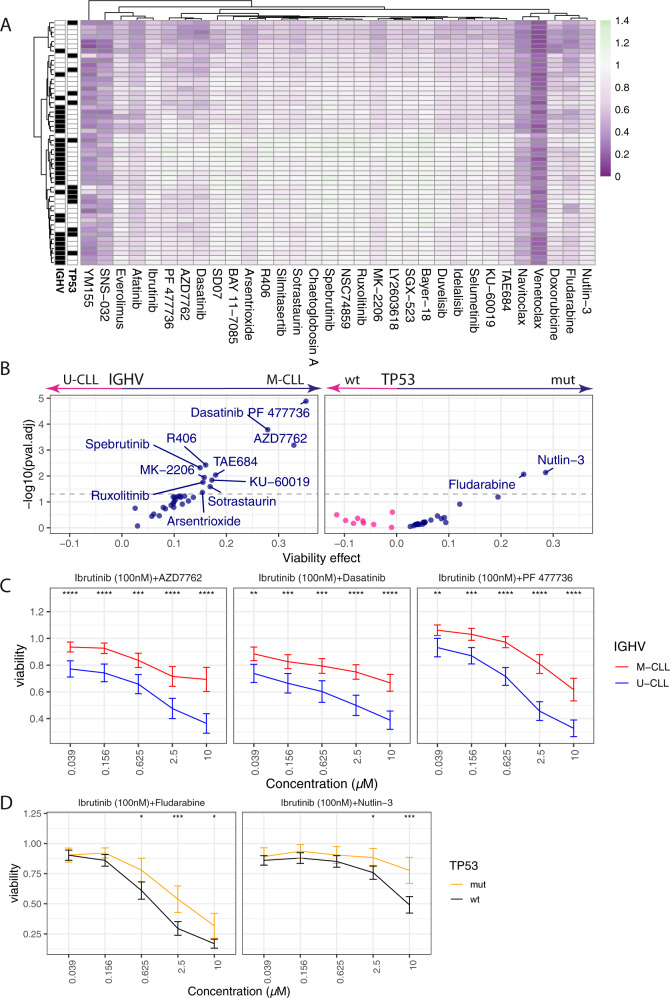
Drug combinations in CLL. **a** Heatmap showing the average viability values in response to the 32 library drugs across 5 concentrations in combination with ibrutinib (100 nM) (*n* = 52). The colors indicate viability values (green: high, purple: low). Hierarchical clustering divides the patient samples into three major subgroups. Distinct response profiles toward combinations of ibrutinib with CHK inhibitors and kinases targeting downstream of the BCR (e.g., MK-2206) separate M-CLL from U-CLL samples. A subgroup of samples (mostly TP53 wild type, IGHV unmutated) shows strong overall sensitivity to most drugs. **b** Volcano plot summarizing differences in response to combinations of ibrutinib (100 nM) with the 32 library drugs based on IGHV (left) and TP53 status (right). The *x*-axis shows the mean difference in the viability values. Positive difference indicates higher sensitivity of U-CLL/TP53 wild type. For each drug, the five concentrations were tested separately and the most significant concentration is shown. The *y*-axis indicates the logarithms of *p* values obtained from a Student’s *t* test and adjusted for multiple testing across both settings and all tested drugs and concentrations. Significant differences were evident for combinations with core BCR-pathway inhibitors (Spebrutinib, R406), CHK inhibitors (PF477736, AZD7762), and dasatinib. Significant differences for TP53 were revealed for fludarabine and nutlin-3 as combination drug. **c** Drug response curves of CLL samples to CHK inhibitors (AZD7762, PF477736) and dasatinib in combination with ibrutinib 100 nM. Shown is the mean viability within each group (M-CLL/U-CLL) and error bars denote two standard errors. U-CLL samples are significantly more sensitive toward CHK inhibitors and dasatinib. *p* values were assessed using Student’s *t* test. **d** Drug response curves as in (**c**) stratified by TP53 mutational status. TP53 wild-type samples are more sensitive toward fludarabine and nutlin-3.

To study heterogeneity of drug responses in the combinatorial screen we first clustered the samples by their response profiles to the 32 agents across concentrations in combination with ibrutinib (*n* = 52) (Fig. 3a, Supplementary Fig. 6). In line with the findings for ibrutinib as a single agent, IGHV status emerged as a dominant factor in the clustering, via several kinase inhibitors (including dasatinib, AZD7762, PF477736). However, using the combinatorial screen we uncovered further heterogeneity of responses. We identified a subgroup of patient samples with high sensitivity to all combinations, which was driven by exquisite sensitivity to ibrutinib (Fig. 3a). These were mainly IGHV unmutated samples (6/7). To further investigate the influence of IGHV status as well as *TP53* mutation on sensitivities toward drug combinations, we tested for such associations using marginal testing (*t*-test) for all drug–drug combinations that had at least three samples in each group (i.e., M-CLL/U-CLL or TP53wt/mut, respectively). As shown in Fig. 3b, the strength of response to a large set of drug combinations with ibrutinib were associated with IGHV mutation status (FDR < 5%), including BCR-pathway inhibitors (spebrutinib, R406), CHK inhibitors (PF477736, AZD7762), and dasatinib (Fig. 3c). Likewise, IGHV status also had a strong impact on drug response phenotypes for combinations with idelalisib (Supplementary Fig 7). These findings are in line with the critical and differential activity of BCR signaling in these molecular groups.

The influence of *TP53* mutations on the response to fludarabine and nutlin-3 as single agents was found to be preserved in combinations with ibrutinib (Fig. 3b, d). In addition, we observed increased effects in the low-programmed methylation cluster for combinations with ibrutinib (*n* = 16) and idelalisib (*n* = 10) with inhibitors of BCR and downstream targets as well as CHK inhibitors as well as for afatinib in combination with CHK inhibitors (Supplementary Fig. 8).

### Landscape of drug combination effects in CLL

**Fig. 4:**
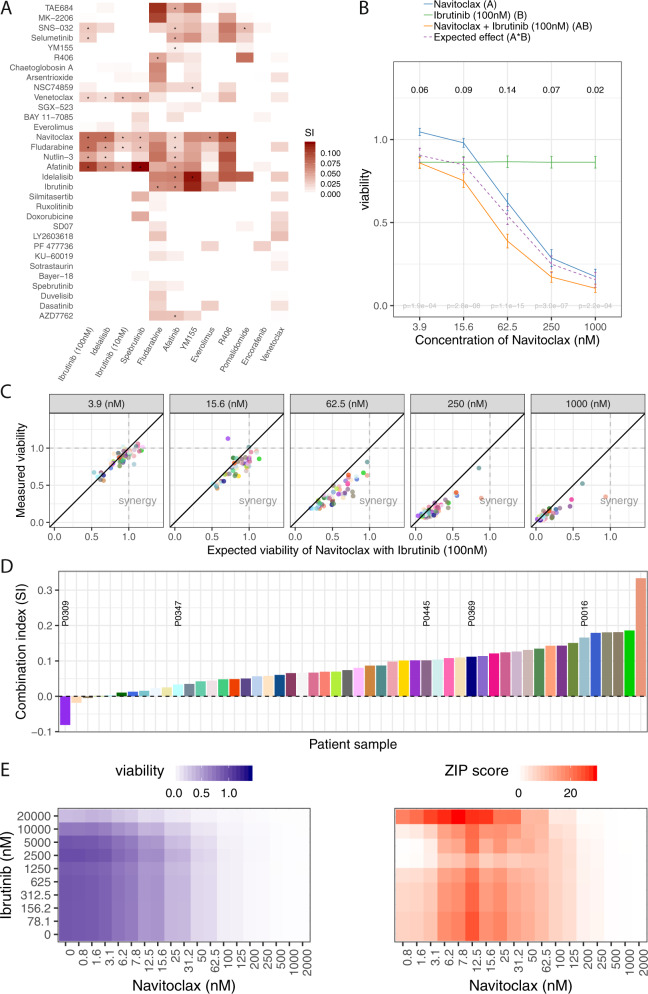
Synergistic interactions in CLL. **a** Heatmap summarizing all synergistic combination effects as determined by the independent effect model (“Material and methods”). Colors indicate for each combination the median synergy index (SI) across all assayed patient samples (numbers depending on the combination drug as in Fig. 1), with red representing synergy (median SI > 0) and white lack of synergy or antagonism (median SI ⩽ 0). Rows and columns are ordered according to a hierarchical clustering dendrogram. Drug combinations with a star showed a significant synergy in at least one concentration at a FDR of 5%. The heatmap revealed combination partners with multiple synergies (synergy index > 0, e.g., navitoclax, fludarabine, afatinib) and showed similarities for different BCR inhibitors in line with the mode of action. Combinations of different BCR inhibitors did not show synergy. **b** Effects of single agents (A, B), measured combination effect (AB) and expected combination effect according to the independent effect model (A*B, “Material and methods”) for navitoclax or ibrutinib alone and their combination is shown. The curves represent the mean viability values across 52 patient samples with error bars denoting two standard errors. The viabilities observed for the combination (AB) were lower compared with the expected effect (A*B), indicating a synergistic effect. Numbers on top indicate this difference for individual concentrations, which was the highest across the concentrations [250–16 nM]; *p* values based on a one-sided paired *t*-test on the differences at each concentration are indicated at the bottom. **c** Scatterplot showing the relationship between measured combination effect AB (viability values on the *y*-axis) and expected combination effect A*B based on the independent effect model (viability values on the *x*-axis) per patient sample (colored dots). Panels correspond to individual concentrations of navitoclax [1 µM–3.9 nM]. The number of synergistic patient samples (SI > 0) at a certain concentration is given by the number of data points below the diagonal (viability values AB < viability values A*B). The synergistic effect is mainly driven by lower concentrations [250–16 nM]. **d** Barplot showing the synergy indices (SI) per patient sample (*n* = 52). Values above 0 indicate synergy (*n* = 49). The labeled patient samples were included in the 10 × 10 validation screen shown in (**e**) (see also Supplementary Fig. 10). **e** Combination activity for navitoclax and ibrutinib in five primary CLL samples as measured in a 10 × 10 matrix of combinational concentration series and assessed using the ZIP score (“Material and methods”). The heatmap indicates the viability values (left) and ZIP scores (right) at each pair of concentrations. ZIP scores shown in the heatmap were smoothed, using the mean ZIP score across the five samples in a running 3 × 3 concentration window. A positive ZIP value (red) denotes synergistic effects, a negative value lack of synergy (blue). Synergistic combination activity is observed in certain concentration ranges.

To gain an overview of combination effects, we clustered the 32 library drugs based on their median synergy indices (“Material and methods”) across patient samples with the 11 combination drugs, including both concentrations for ibrutinib (Fig. 4a). We identified drugs with multiple synergies (e.g., fludarabine (*n* = 5 out of 12), nutlin-3 (*n* = 3 out of 12), navitoclax (*n* = 7 out of 12), venetoclax (*n* = 4 out of 12), and afatinib (*n* = 5 out of 12)) and found striking similarities for combinations involving a BCR inhibitor as one of the partners, which suggests strong functional convergence (Fig. 4a). For instance, both ibrutinib and idelalisib had similar, strong patterns of synergy with the Bcl-2 inhibitor navitoclax. In contrast, combinations of different BCRi or of BCRi and the CHK inhibitors did not display synergy (white, SI ⩽ 0). While it might be hypothesized that simultaneous inhibition of multiple BCR kinases (BTK, PI3K, SYK) could provide synergy compared with single target inhibition [22, 29], e.g., due to increased efficacy of BCR inhibition, our results indicate that this is not the case. Simultaneous inhibition of downstream pathway members (AKT, mTOR) did not result in synergy according to the synergy index but led to an additional viability decrease compared with the effect of the single drugs (Supplementary Fig. 9). In particular, combination of the mTOR inhibitor everolimus with ibrutinib resulted in decreased viabilities compared with each single drug (78–81% mean viability of control across 5 concentrations for combination vs. 86% mean viability for ibrutinib 100 nM vs. 91–94% mean viability for everolimus) (Supplementary Fig. 9). Similarly, combination of ibrutinib with the AKT-inhibitor MK-2206 showed decreased viability compared with the single drugs: mean viability under the combination ranged from 58–85%, compared with 63–101% for MK-2206 alone and 86% for ibrutinib 100 nM alone (Supplementary Fig. 9). These findings imply that AKT and mTOR are activated in part in a BCR independent manner in CLL, and thus are relevant targets even in the presence of BCRi.

### BCRi and Bcl-2 inhibitors show synergy in CLL

Navitoclax is a Bcl-2/Bcl-X_L_/Bcl-w inhibitor with clinical activity in CLL [47] and recent clinical studies [30, 31] suggest that combinations of BH3 mimetics and inhibitors of BCR signaling are effective in CLL. Indeed, we observed strong cooperative activity of navitoclax in combination with ibrutinib (100 nM), predominantly at intermediate concentrations of navitoclax [2.5 µM–15.6 nM] (Fig. 4b). To understand inter-individual differences between such synergistic effects, we considered the individual patient samples’ SIs (Fig. 4c, d). Interactions were synergistic (SI > 0) in almost all patient samples (49/52), with a median of 0.08 (Fig. 4d). The SI was correlated with sensitivity to navitoclax as single agent with high synergy indices for less sensitive samples. Using a 10 × 10 combination matrix of concentration series (“Material and methods”, Fig. 4e, Supplementary Fig. 10) we confirmed synergistic effects across concentration ranges (strongest for navitoclax 6.2–50 nM). At higher concentrations of navitoclax no synergy could be observed due to complete cell death already under the single agent.

As thrombocytopenia caused by Bcl-X_L_ inhibition limited its clinical use [48], venetoclax, a selective Bcl-2 inhibitor, was developed [49]. Venetoclax showed stronger viability effects than navitoclax at equimolar dose [49] [1 µM–250 nM] (Supplementary Fig. 11). Therefore, synergistic activity in combination with ibrutinib 100 nM was observed for doses below 63 nM. We confirmed the synergistic effects of venetoclax and ibrutinib across concentration ranges (strongest for ibrutinib 78 nM–1250 M, venetoclax 1.56–6 nM) using a 10 × 10 concentration matrix (Supplementary Fig. 11E).

In summary, these results confirm that combinations of BH3 mimetics and inhibitors of BCR signaling are effective in CLL, supporting recent clinical studies [30, 31] and current trial activity (NCT02756897, NCT03580928, NCT03868722).

### Combinations of BCRi with fludarabine or nutlin-3

Fludarabine-based combinations are standard of care in CLL [50]. Previous work suggested combination activity of ibrutinib and chemotherapeutic agents such as the purine analog fludarabine [51]. Our screen uncovered a number of potentially synergistic combinations for fludarabine including BCR inhibitors (Fig. 4a). Ibrutinib and fludarabine showed synergistic effects across multiple concentrations (median SI: 0.08) (Supplementary Fig. 12). This effect was observed in 48/52 patient samples. Based on the association of p53 status and IGHV on the effect of both drugs, we asked how *TP53* and IGHV status influenced synergy. While unmutated IGHV positively influenced synergy, *TP53* status did not influence synergy (Supplementary Fig. 12D, E).

We observed synergistic combination activity of the Mdm2 inhibitor nutlin-3 with ibrutinib, idelalisib, spebrutinib, and R406 (Fig. 4a). This includes the combination of ibrutinib 100 nM with higher concentrations of nutlin-3 (10–2.5 µM), with a median SI of 0.05 and SI > 0 in 42/52 patient samples (Supplementary Fig. 13), in line with a finding of Voltan et al. [26].

### Combinations of BCRi and afatinib display synergy in CLL

**Fig. 5:**
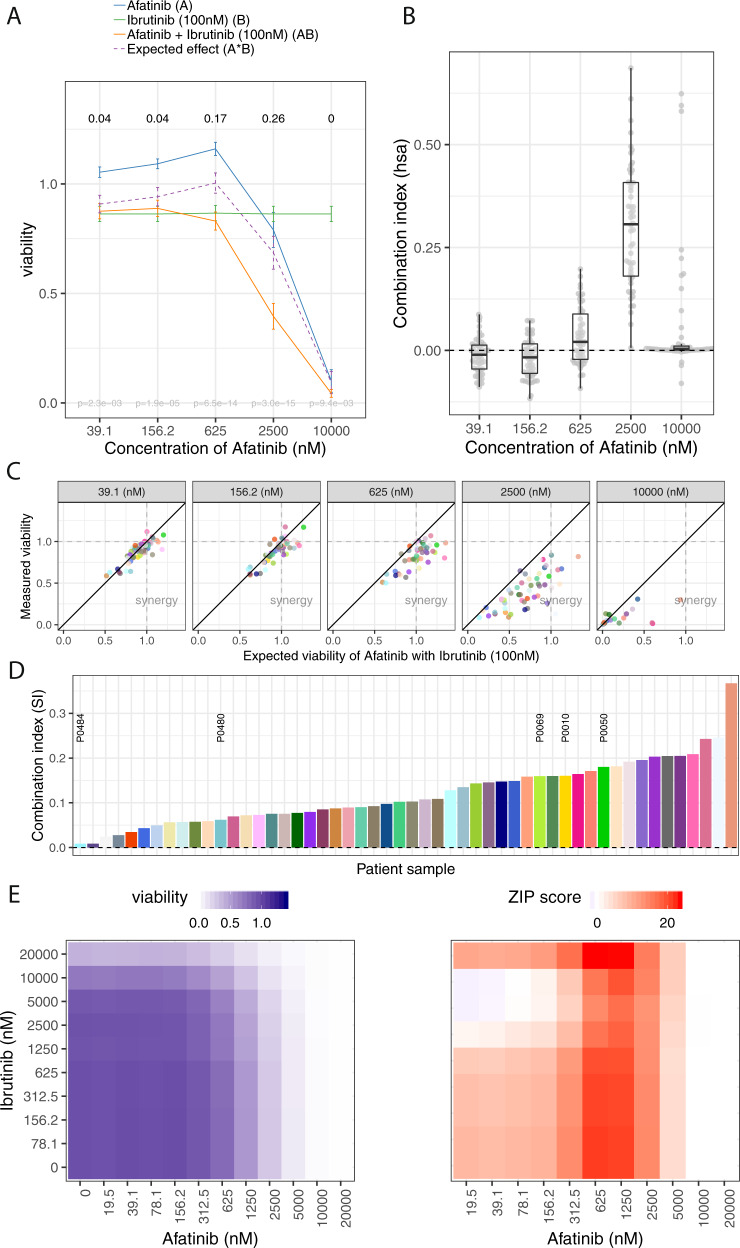
Synergy of afatinib and ibrutinib. **a** Effect of single agents (A, B), measured (AB) and expected combination effects (A*B, calculated based on the independent effect model) in 52 patient samples for afatinib and ibrutinib. Drug response curves represent the mean viability values at each concentration, error bars denote two standard errors. The strongest synergistic combination effects were observed at intermediate concentrations [2.5 µM–625 nM]. **b** Boxplots showing the combination index (CI) across patient samples as determined by the highest single agent approach for afatinib and ibrutinib for five concentrations [10 µM–39 nM]. Synergistic effects can be observed in 2.5 µM–625 nM. **c** Scatterplot showing the relationship between measured combination effect AB (viability values on the *y*-axis) and expected combination effect A*B based on the independent effect model (viability values on the *x*-axis) per patient sample (colored dots). Panels correspond to individual concentrations of afatinib [10 µM–39 nM]. The number of synergistic patient samples (SI > 0) at a certain concentration is given by the number of data points below the diagonal (AB < A*B). **d** Synergy indices as determined by the independent effect model per patient sample (*n* = 52). All patient samples showed synergy (*n* = 52); the median synergy index was 0.17. Labeled patient samples were included in the 10 × 10 validation experiment shown in (**e**) (see also Supplementary Fig. 17). **e** Combination activity for afatinib and ibrutinib as visualized by a 10 × 10 matrix of combinational concentration series as in Fig. 4e.

Afatinib is an EGFR inhibitor in clinical use for nonsmall cellular lung cancer harboring activating EGFR mutations [52]. A striking number of synergistic interactions were observed for afatinib in combination with BCRi (Fig. 4a). For example, afatinib in combination with ibrutinib or spebrutinib showed the highest SI among all combinations with afatinib (0.10 for ibrutinib 100 nM; 0.12 for spebrutinib). To understand this unexpected finding, we assessed the effect of afatinib across concentrations and observed that afatinib increased viability at low concentrations (Fig. 5a). Ibrutinib completely antagonized this effect, suggesting that the effect is mediated by interference with upstream parts of BCR signaling. The synergistic effect was most pronounced at intermediate afatinib concentrations [2.5 and 625 nM] (Fig. 5a–c). Synergy indices calculated individually for each patient sample revealed synergy for all patient samples (Fig. 5d) with a tendency of higher synergy in U-CLL samples (Supplementary Fig. 14).

To expand on these findings, we included afatinib as a combination drug and assayed it against the 32 drug library for 30 samples. Synergy with BCRi including ibrutinib, idelalisib, and R406 (Supplementary Fig. 15) and the abolition of afatinib’s prosurvival effects by ibrutinib were confirmed (Supplementary Fig. 16). The synergistic effect of combinations of ibrutinib with afatinib at most concentrations (strongest for afatinib 625 nM–1.25 µM) was also verified using 10 × 10 concentration matrices starting at 5 µM (three patient samples) and 20 µM (two patient samples) (Fig. 5e, Supplementary Fig. 17).

To further confirm the effects exerted by afatinib we investigated the viability effects by FACS (Annexin/7AAD). We observed increased viability after 24 and 48 h, an effect blocked by addition of ibrutinib (data not shown). Afatinib was designed to inhibit EGFR at high specificity. Its effect in our study, however, is unlikely to be mediated via EGFR, as EGFR is not expressed in CLL cells [53]. We also found no evidence for the expression of EGFR in CLL cells at RNA or protein level (data not shown). Further binding partners of afatinib include ERBB2, ERBB4, GAK, BLK, IRAK1 [54]. To explore the relevant target(s) in CLL, we analyzed RNA expression data, which showed that BLK, GAK, IRAK1a, and MAPK9/14 are expressed in CLL; thus it is possible that the activity of afatinib in CLL is mediated via one or more of those components (Supplementary Fig. 18A). Irreversible kinase inhibitors such as afatinib and ibrutinib, which target a cysteine residue in the ATP binding site of kinases such as EGFR and BTK, often bind both, EGFR and BTK, albeit often with higher potency for the intended target [55]. When testing other EGFR inhibitors, we found for some of them (e.g., canertinib) similar dose–response curves including a prosurvival effect at lower or intermediate concentrations (Supplementary Fig 19). Previous work showed that EGFR inhibitors in AML cells can target Syk within the BCR pathway and induce cell differentiation and cell death [56–58]. We used the PamGene array (“Material and methods”) to study the effect of afatinib on kinase activity in three primary CLL samples sensitive to afatinib treatment. By adding afatinib before or after lysis of the cells we identified multiple members of the Src-Kinase family (BLK, SRC, YES) as well as the Syk kinase as direct and downstream targets of the drug (Supplementary Fig 18B). When testing the combination activity of ibrutinib with a Syk inhibitor (R406) we did not find synergy (Supplementary Fig 20). In summary, inhibition of Syk in CLL does not fully explain the prosurvival effect of afatinib and abolition of this effect when adding ibrutinib. This indicates that afatinib effects in CLL might be mediated by BLK functioning upstream of BCR-signaling components such as BTK.

### Synergy of ibrutinib and MEK-inhibitor selumetinib

The effectiveness of MEK inhibition in CLL has been demonstrated in ex vivo studies [59]. Our screen uncovered a synergistic combination activity of ibrutinib and selumetinib, predominantly at higher (>625 nM) selumetinib concentrations (Supplementary Fig. 21). While the overall effect was weak, it was consistently observed in 39/52 patient samples (median SI: 0.02), suggesting that a part of MEK activation in CLL is independent of the BCR.

### Combination of BCR inhibitors lack cooperative activity in CLL

**Fig. 6:**
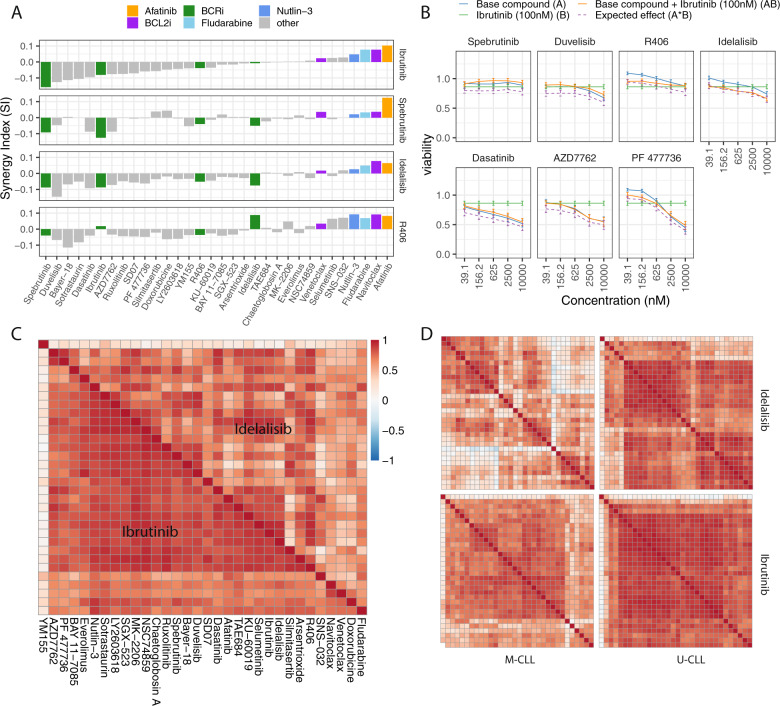
BCR inhibitors in combination. **a** Median synergy indices (SI, “Material and methods”) across all available patient samples for BCRi including ibrutinib, idelalisib, spebrutinib, and R406 in combination with the 32 drug library. Values above 0 indicate synergy. Numbers of patient samples available for the combination drugs were 52 (ibrutinib), 30 (idelalisib), 16 (spebrutinib), and 6 (R406). Combination with BCRi resulted in cooperative effects with afatinib, navitoclax, fludarabine, and nutlin-3. Combinations among BCRi did not show synergy, except idelalisib or ibrutinib in combination with R406 in six patient samples. **b** Curves show the mean viability values in response to selected library drugs (BCRi or CHK inhibitors/dasatinib) as single agent or in combination with ibrutinib 100 nM across 52 CLL patient samples. Comparable cytotoxic effects were observed across concentrations for the library drugs as single agents and in combination. **c** The heatmap shows the correlations of the 32 library drugs in combination with ibrutinib (lower triangle) and idelalisib (upper triangle) based on the viability values across 30 patient samples. Clusters of drugs with high correlation appear as red squares. Combining the library drugs with ibrutinib lead to overall increased correlation between pairs of library drugs than for the library drugs alone (Fig. 2e), for idelalisib this effect was weaker. **d** As in (**c**) shown separately for U- and M-CLL. Correlation of ibrutinib and idelalisib was similar within U-CLL. For M-CLL, differences specific to idelalisib were observed.

To characterize potential cooperative effects among different inhibitors of the BCR pathway, we included inhibitors of BTK (ibrutinib, spebrutinib), PI3K (idelalisib), and Syk (R406) in the drug library. Combination of these BCRi with one another did not result in synergistic effects. As shown in Fig. 6a, the BCRi combinations showed small or no synergistic effects. For example, combination of ibrutinib with spebrutinib, and of idelalisib with duvelisib showed the lowest SI, in line with complete target inhibition by either drug. The use of BCR and CHK inhibitors or dasatinib lacked cooperative combination activity (51–89% median viability in combination vs. 53–86% for AZD7762 alone, 50–83% in combination vs. 48-81% for dasatinib alone, 46–102% in combination vs. 42–109% for PF477736 alone), suggesting that the activity of all these inhibitors in CLL derives from already maximal BCR component inhibition (Fig. 6b).

### Combinations of BCR inhibitors with other drugs

Given the overall similarity of combination effects of BTK and PI3K inhibitors (Fig. 4a), we asked whether there may also be differences between them. We exploited the parallel design of our study to compare combinations involving idelalisib and combinations involving ibrutinib. In line with co-targeting of the BCR we found highly correlated patterns of interactions with the drug library (*r* = 0.94). However, we observed drug-specific differences of the combination effects, suggesting that the effects of inhibition of BTK and PI3K are not identical (FDR < 5%, paired *t*-test for each concentration, Supplementary Fig. 22). In particular, we observed differential sensitivities for the combination with BTK inhibitor spebrutinib, where combinations with the PI3K inhibitor idelalisib were significantly more effective, and for the combination with afatinib, where combinations with ibrutinib 100 nM were significantly more effective (Supplementary Fig. 22). These differences were unlikely to be caused by dosage, as ibrutinib and idelalisib had similar cytotoxicity at 100 nM (median viability across all combinations: 84.3% for ibrutinib 100 nM vs. 83.8% for idelalisib 100 nM, *p* = 0.68). Taking a global view across all library drugs, we found that combination with ibrutinib resulted in a stronger correlation between the response profiles of the different library drugs compared with idelalisib (Fig. 6c, red cluster). These differences were more pronounced in M-CLL than in U-CLL (Fig. 6d).

## Discussion

While inhibitors of the BCR have revolutionized treatment options, addressing resistance and relapse requires systematic approaches for identifying effective drug combinations. Here we present an unbiased screen to test drug combinations in CLL and use it to identify synergistic drug combinations from a panel of 32 × 11 drugs. We identified novel auspicious therapeutic options by co-targeting the BCR pathway with other pathway dependencies and observed striking combination activity of BCRi with a range of mechanistically diverse compound classes including Bcl-2 inhibitors, chemotherapeutic agents, and afatinib. Bcl-2 inhibitors were previously suggested by ex vivo and in vivo studies to show beneficial combination effects with ibrutinib in CLL, DLBCL, and MCL [22, 28, 30, 31, 60–62]. This synergistic effect was confirmed in our screen for two Bcl-2 inhibitors, navitoclax, and venetoclax, and can be explained by simultaneous interruption of two pathways representing salvage pathways under monotherapy [28]. The doses of ibrutinib, idelalisib, venetoclax, and navitoclax used in our experiments are attainable in patients’ blood plasma [11, 28, 49], so translation of such combinations into the clinic appears feasible. Indeed, the first clinical studies in patients undergoing combination therapy showed an acceptable tolerance and a similar adverse-event profile compared with monotherapy with ibrutinib or venetoclax [30, 31].

Furthermore, our screen recovered synergistic activity of ibrutinib and idelalisib in combination with fludarabine. This is in line with previous preclinical work that suggested combination activity of chemotherapeutic agents with ibrutinib in DLBCL by disruption of NF-*κ*B signaling and decreased expression of antiapoptotic proteins by ibrutinib re-sensibilizing B-cells to undergo apoptosis [22]. In CLL, a sensitization of idelalisib treated CLL cells to fludarabine was described [15]. In line with preclinical data, clinical studies suggested beneficial combination effects of chemotherapeutic agents with ibrutinib in CLL [34, 35]. In addition, we observed cooperative combination activity for MEK-inhibitor selumetinib and ibrutinib. This complements previous studies in DLBCL and MCL cell lines and a DLBCL mouse model that suggested potentiation of ibrutinib’s viability decreasing effects by downregulation of p-ERK-1/2 through inhibition of the MEK/ERK/AKT pathway [63].

Interestingly, synergistic effects were also observed for EGFR inhibitor afatinib in combination with BCRi as well as with agents targeting downstream (e.g., TAE684, NSC74859, MK-2206). Previous work demonstrated that the activity of EGFR inhibitor gefitinib in U-CLL by inhibition of BCR signaling via reduced phosphorylation of Syk/Zap-70/ERK/AKT and decreasing prosurvival proteins such as Mcl-1 [53]. Likewise, in AML Syk inhibition through gefitinib suggests an EGFR independent mode of action in leukemia cells [56–58]. The role of Syk is supported by the PamGene kinase assay that we performed in CLL cells with afatinib, where we found Syk among the top ten targets of afatinib. For afatinib, synergistic effects resulted in part from abolition of its prosurvival effects at low concentrations. Here, we identified BLK as a possible afatinib target upstream of the BCR. This connection to the BCR might explain the synergistic effects.

Although previous studies demonstrated that the efficacy of combinations of BCRi in DLBCL cell lines, CLL and MCL [22, 29, 61, 64], we observed no evidence of beneficial combination effect for ibrutinib with other BCRi in our study. Nevertheless, targeting downstream elements in the BCR pathway could result in increased cytotoxicity in vivo as BCRi induce mobilization of CLL cells from the lymph nodes, their survival promoting niches in vivo [29]. While the combination of 32 drugs with ibrutinib or idelalisib suggested overall similarity based on targeting the BCR, we uncovered pathway-specific differences. These were most pronounced in M-CLL where BCR signaling is less crucial and several other survival pathways are important [65–67]. Here, the effect of idelalisib on correlation of drug combinations was less pronounced compared with ibrutinib. This might be due to idelalisib targeting downstream of ibrutinib and PI3K being involved in many other pathways compared with BTK.

While this screening platform offers a practical systematic approach for the identification of new drug combinations in primary leukemia cells ex vivo, it also has limitations. Cell death was assessed using an ATP-based viability assay (CellTiter Glo), therefore agents with negligible cytotoxicity cannot be judged well. CLL cells taken from PB do not proliferate, so the effect of drugs impeding proliferation cannot be gauged [28]. Preclinical ex vivo models can also not model organismal toxicity, as evidenced by unexpected severe organ toxicities of combination approaches in clinical trials. Due to a limited sample size, our screen lacked the power to uncover further heterogeneity of drug effects due to molecular heterogeneity of tumors. While we did not incorporate VAFs into the analysis, this information could be used to further increase the power to detect response differences associated with somatic mutations.

Despite these limitations, this study identified potentially promising combination approaches for CLL that may warrant translation into clinical trials. In particular, this study, supported by the prior results [30, 31, 34, 35], provides a rationale for further trialing the addition of Bcl-2 inhibitors or fludarabine to ibrutinib/idelalisib therapy. Second, given the striking synergistic interaction of BCRi with the EGFR inhibitor afatinib, activity of afatinib alone and in combination with BCRi in CLL should be further assessed.

To foster further research in this area, we provide a Shiny web resource as a resource for the community to explore the results from our screen (http://mozi.embl.de/public/combiScreen).

## Supplementary information

Supplementary figures

## Data Availability

The data of the molecular profiling were obtained from Dietrich et al. [37]. The raw data generated by the combinatorial drug screen experiments are available from the EMBL-EBI BioStudies repository (accession number S-BSST381). The processed data can be explored using our Shiny App http://mozi.embl.de/public/combiScreen.

## Code availability

The code for the analysis contained in this paper is available at https://github.com/bv2/drug_combi_screen_CLL.
