# Supplementary material for: Survey of ex vivo drug combination effects in chronic lymphocytic leukemia reveals synergistic drug effects and genetic dependencies
Source: Leukemia. 2020 May 13;34(11):2934–50. doi: 10.1038/s41375-020-0846-5 (PMC7584477; doi:10.1038/s41375-020-0846-5)
Supplement: Supplementary file 1 — Supplementary figures [file 41375_2020_846_MOESM1_ESM.pdf]

Supplementary figures

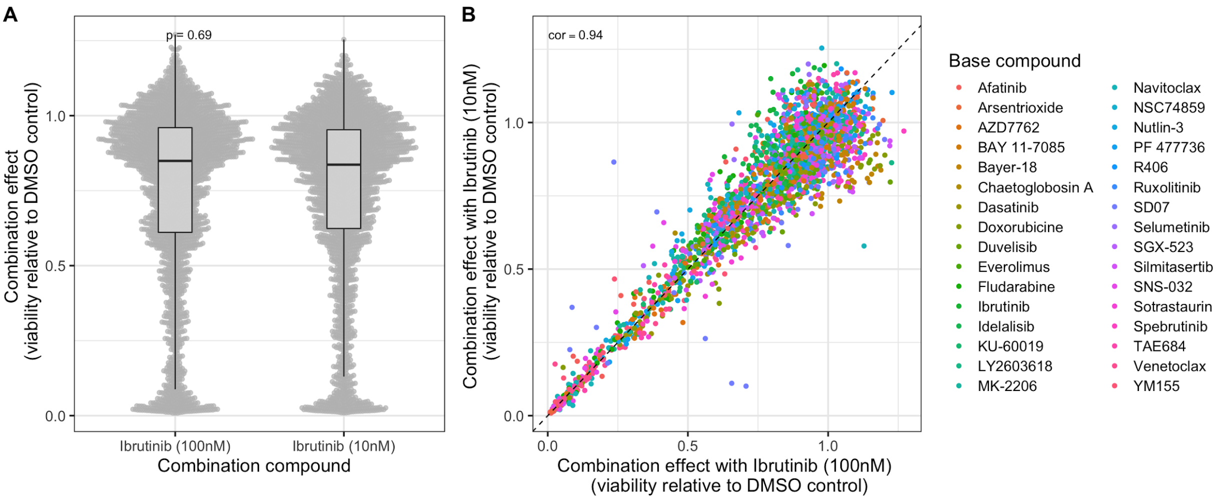

Suppl. Fig. 1: Ibrutinib 100 nM vs. 10 nM

A) Beeswarm plot comparing the viability values in response to combinations with ibrutinib 100 nM and ibrutinib 10 nM in 16 patient samples and a total of 2554 measurements. The cytotoxicity effect (median of 83.7% vs. 84.9% viability relative to control) was not significantly different (p=0.69).

B) Scatter plot comparing the viability values in response to the 32 library drugs in combination with either ibrutinib 100 nM or ibrutinib 10 nM across all 5 concentrations in 16 patient samples. Overall correlation was r=0.94.

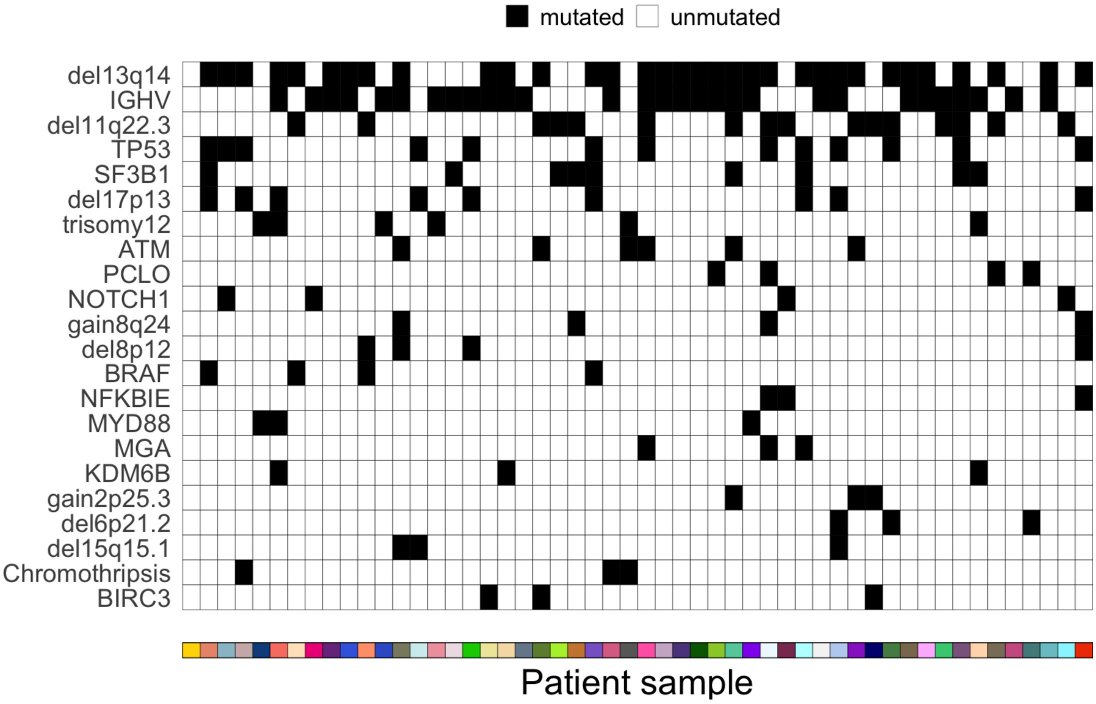

Suppl. Fig. 2: Overview of patient cohort and their genetic heterogeneity

Genetic background of the *n*=52 patient samples. Each column corresponds to an individual patient sample, rows show main genetic aberrations, i.e. those present in at least three samples.

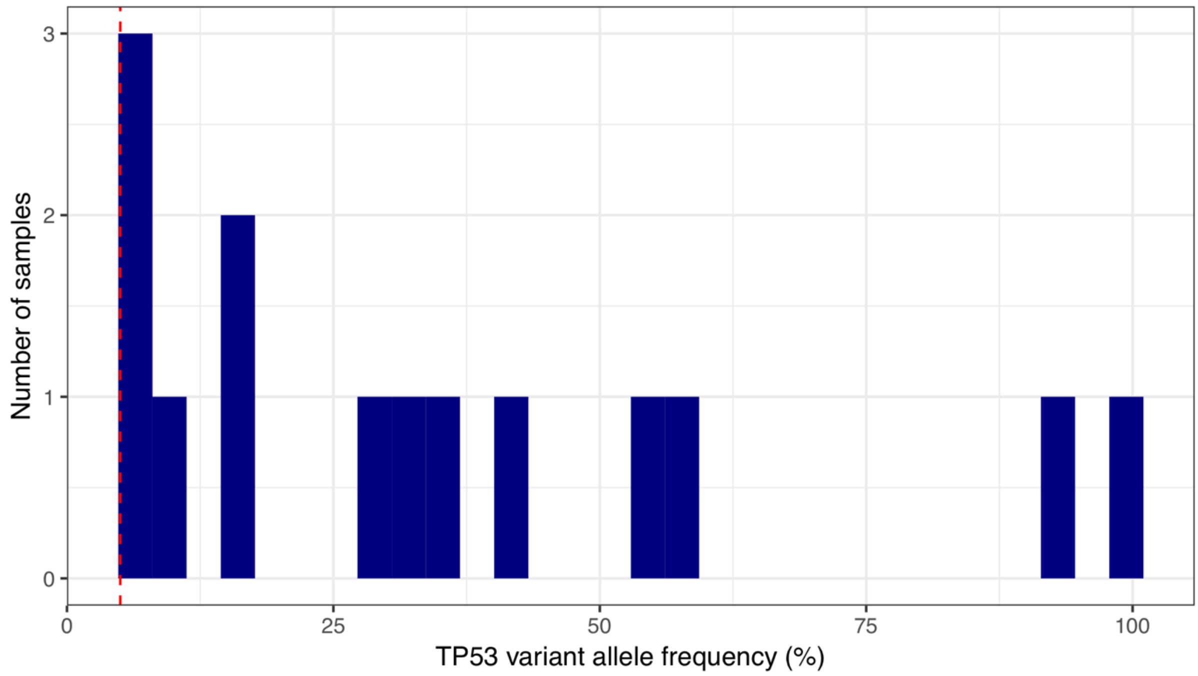

Suppl. Fig. 3: Variant allele frequency of TP53
Histogram of variant allele frequencies (VAF) of TP53 the *n*=52 patient samples. Bars indicate number of samples (y-axis) with a specific VAF (x-axis). The vertical red line indicates 5%.

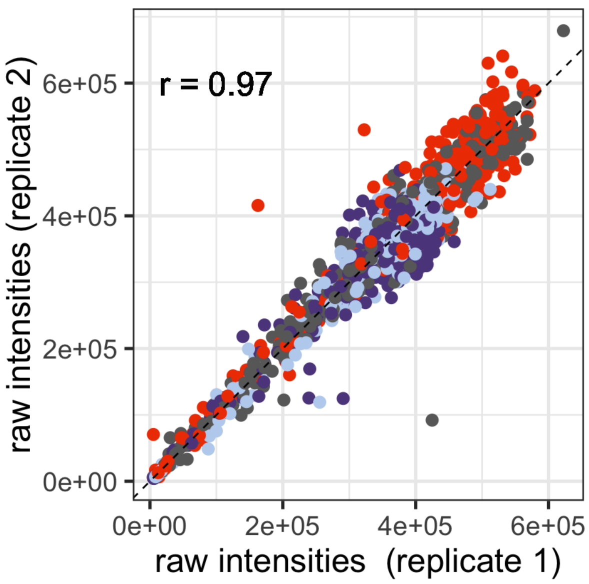

Suppl. Fig. 4: Replicate measurements demonstrate reproducibility

Scatter plot showing raw intensity values across all drug combinations and concentrations for two replicates. Patient samples (n=4) are indicated in different colors, and the measurement pairs are plotted on the x- and y-axis. The overall correlation is r=0.97.

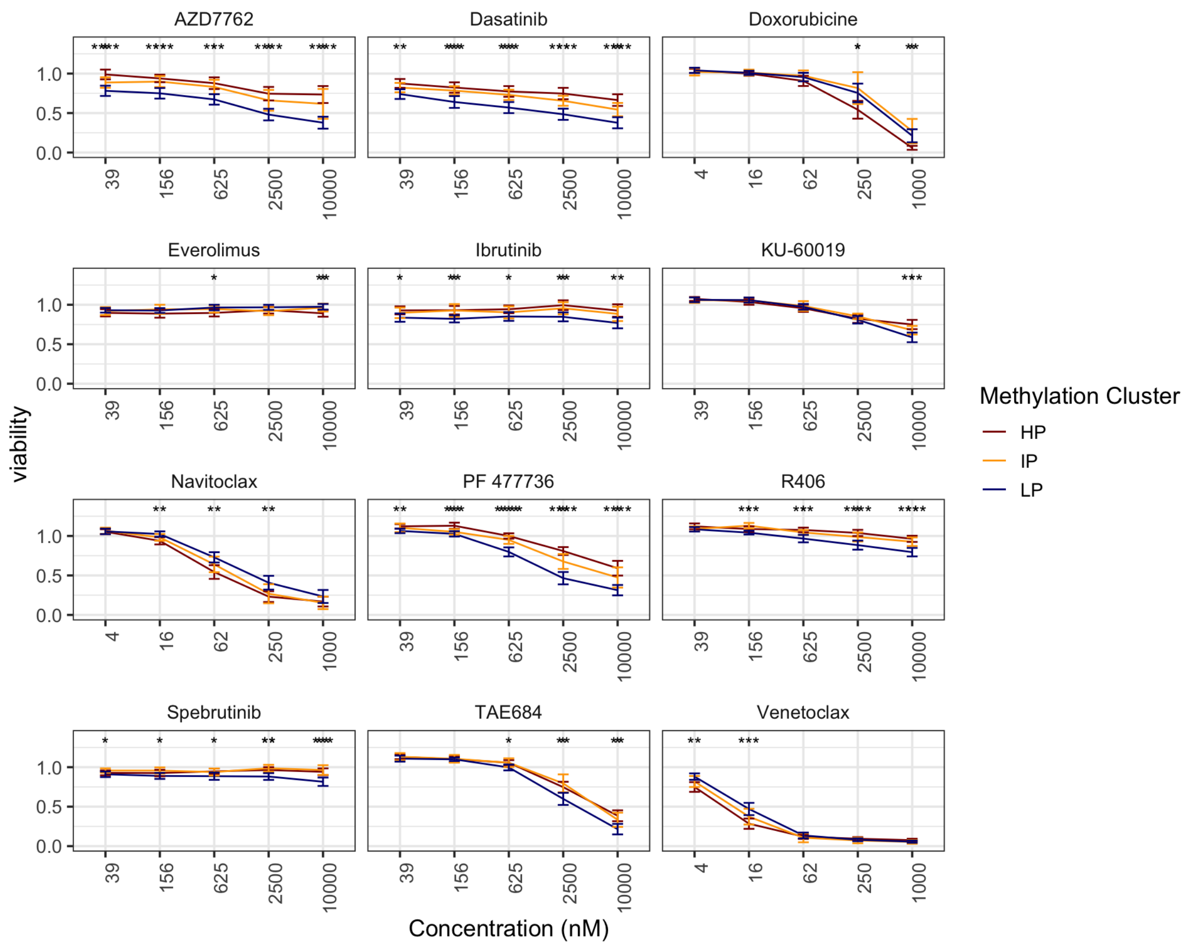

Suppl. Fig. 5: Impact of methylation cluster on response to single agents

Drug response curves stratified by methylation cluster for single drugs that showed a significant association to methylation cluster groups. LP denoted low-programmed, IP intermediate-programmed and HP high-programmed cluster as in Oakes et al, 2016. Significance was assessed by an F-test in a linear model for individual concentrations (*: p <= 0.05, **: p <= 0.01, ***: p <= 0.001,****: p <= 0.0001).

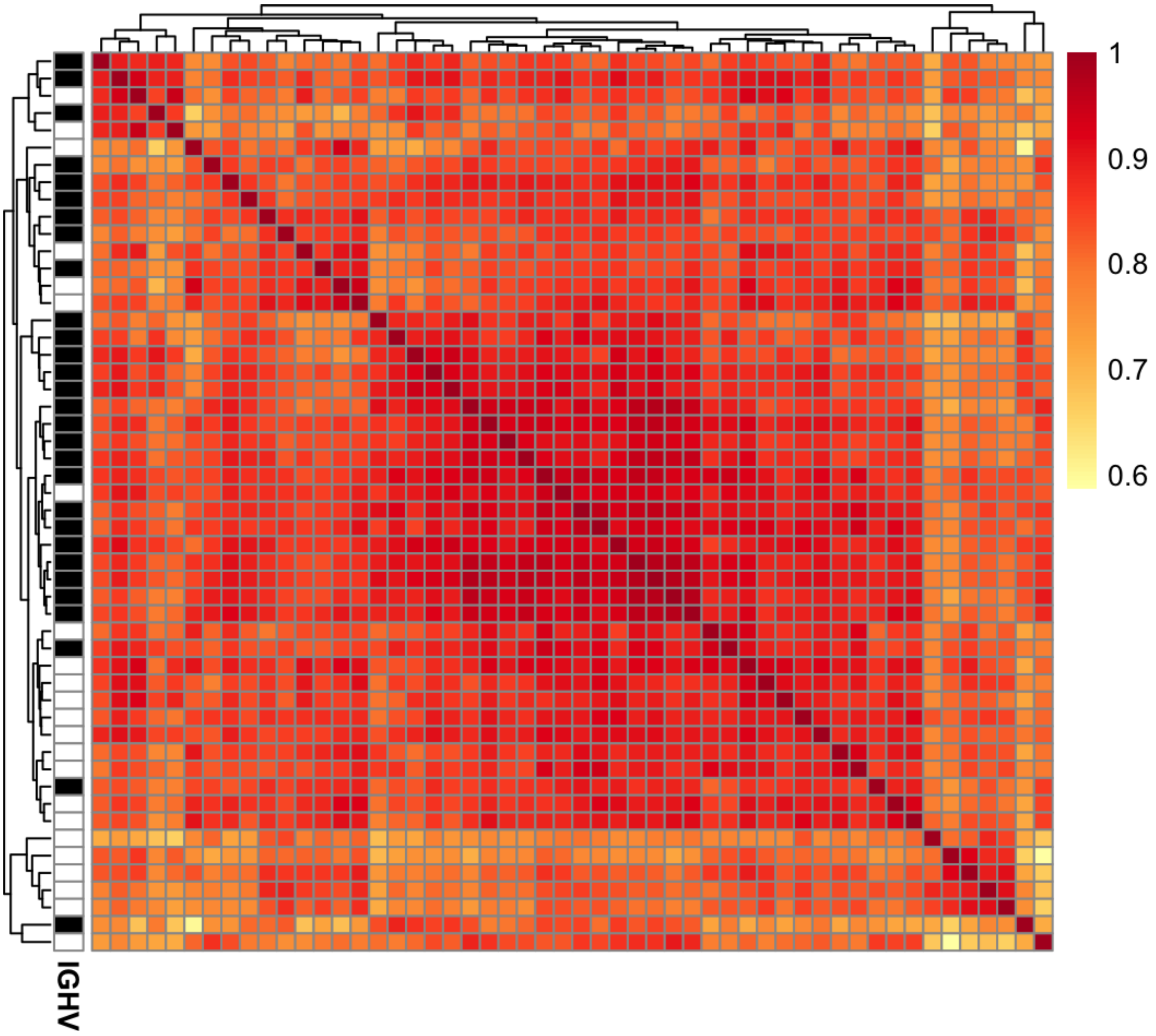

Suppl. Fig. 6: Patient sample correlation matrix

Heatmap shows sample-sample correlations indicating the similarity of the 52 patient samples' responses towards combinations with ibrutinib 100 nM. M-CLL samples are annotated in black. Based on clustering, distinct patterns of high correlation emerged for U-CLL and M-CLL samples.

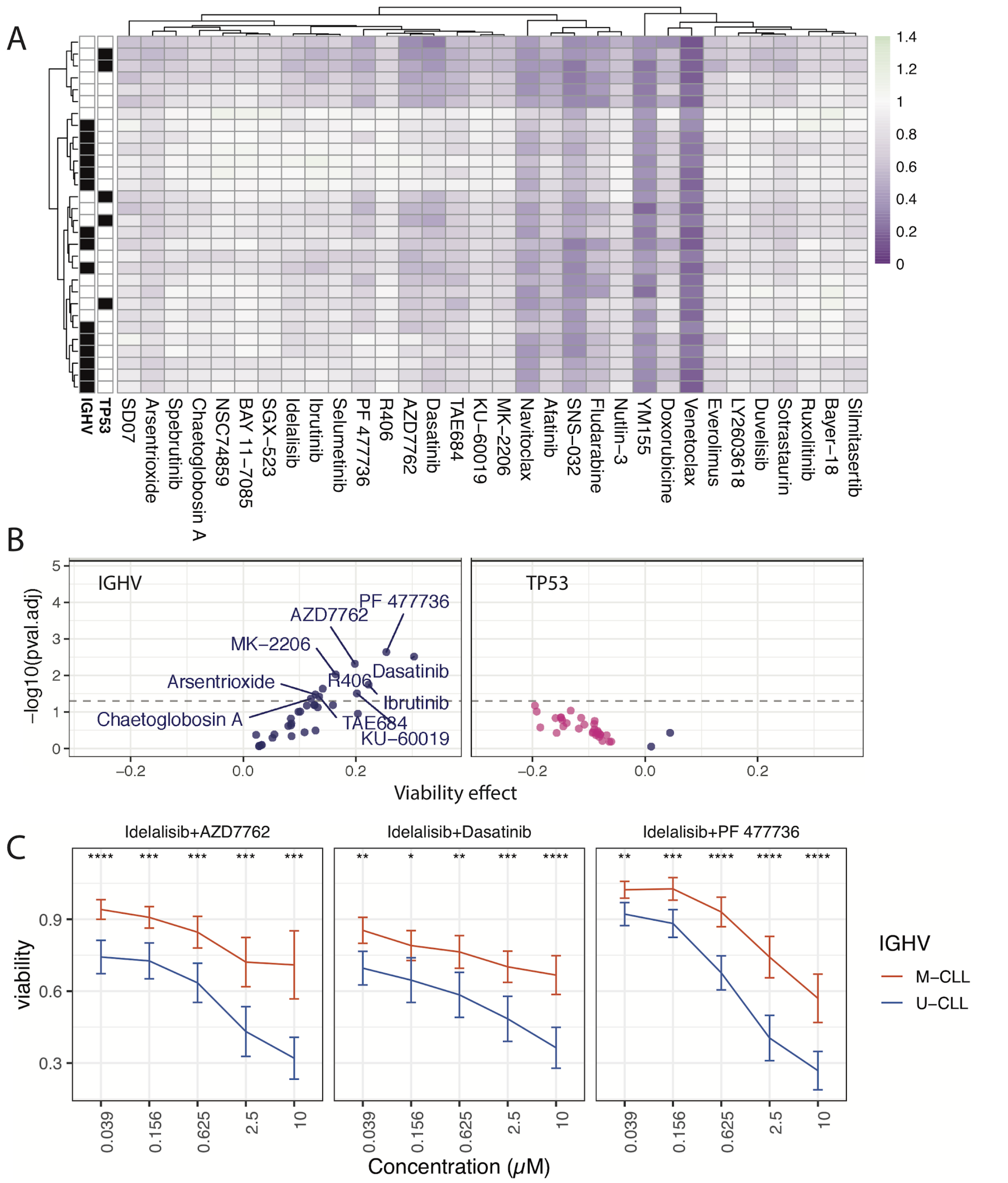

Suppl. Fig. 7: Patient sample specific drug effects for combinations with idelalisib

A) Heatmap showing drug responses towards 32 drugs in combination with idelalisib in 30 patient samples. Color codes display viability values as in **Fig. 3A**. Distinct response profiles towards combinations of idelalisib with CHK inhibitors, BCRi and kinases targeting downstream of the BCR (e.g. MK-2206) separate M-CLL from U-CLL samples.

B) Volcano plot summarizing differences in response to combinations with idelalisib (100 nM) between U-CLL and M-CLL (left) and TP53 wildtype and mutated samples (right). Mean difference in the viability values is shown on the x-axis. Positive difference indicates higher sensitivity of U-CLL/TP53 wildtype compared to M-CLL/TP53 mutated. For each drug, the five concentrations were tested separately and the most significant concentration is shown. Significant differences were evident for combinations with core BCR pathway inhibitors (ibrutinib, R406), CHK inhibitors (PF 477736, AZD7762) and dasatinib. Significance was tested using the Student's *t*-test. The logarithms of the multiple-testing adjusted p-

values (across both molecular markers, all drug-drug combinations and concentrations) are indicated on the y-axis.

C) Drug response curves of CLL samples to CHK inhibitors (AZD7762, PF477736) and dasatinib in combination with idelalisib. Shown is the mean viability within each group (M-CLL/U-CLL) and error bars denote two standard errors. U-CLL samples are significantly more sensitive towards the combination with CHK-inhibitors and dasatinib. P-values were assessed using Student's t-test. (*: p <= 0.05, **: p <= 0.01, ***: p <= 0.001,****: p <= 0.0001)

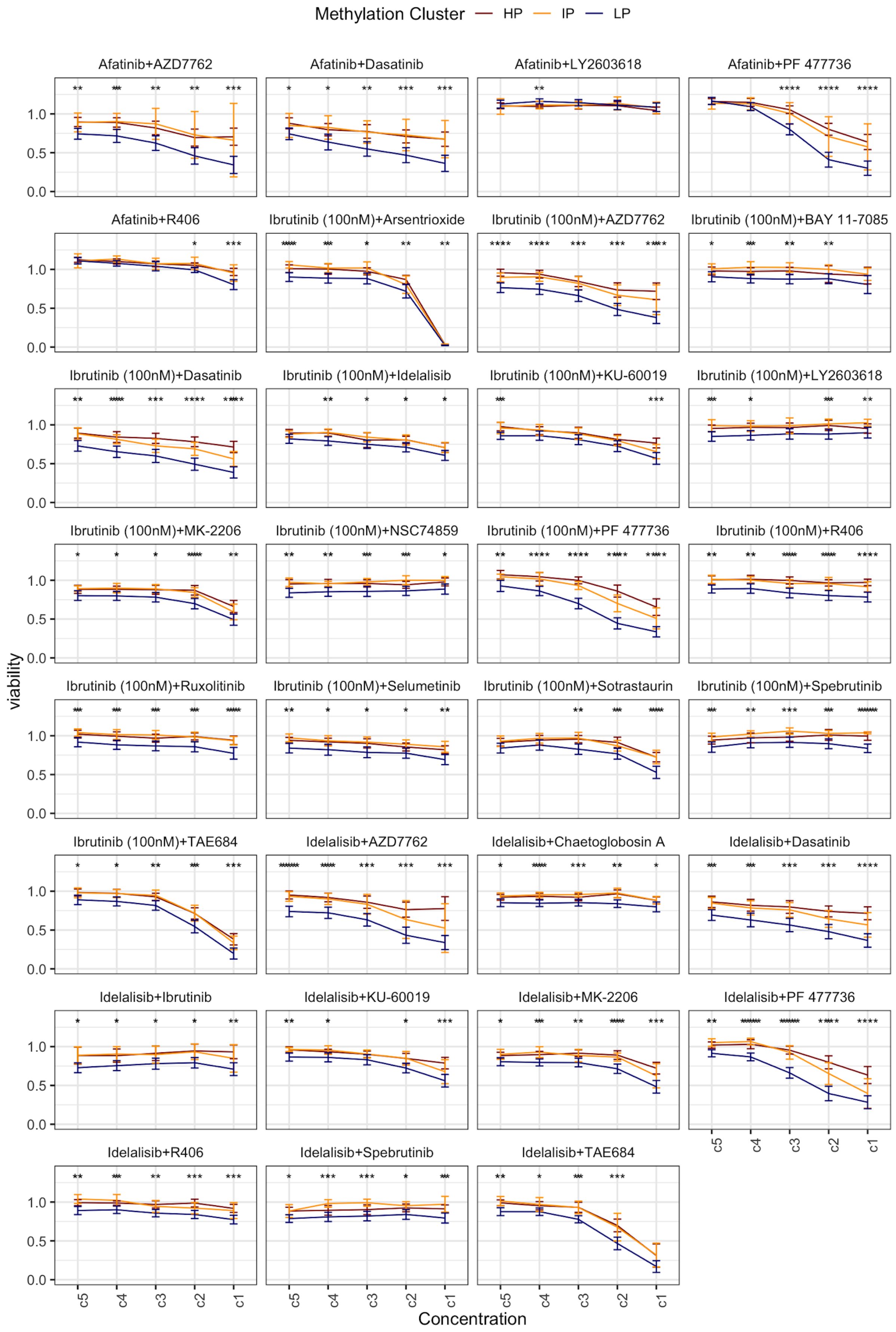

Suppl. Fig. 8: Impact of methylation cluster on response to drug combinations

Drug response curves stratified by methylation cluster for drug combinations that showed a significant association to methylation cluster groups. Shown is the mean viability within each group, error bars denote two standard errors. LP denoted low-programmed, IP intermediate-programmed and HP high-programmed cluster as in Oakes et al, 2016. Significance was assessed by an F-test in a linear model relating viability values to the methylation cluster group for individual concentrations (*: p <= 0.05, **: p <= 0.01, ***: p <= 0.001,****: p <= 0.0001). Only a single ibrutinib concentration is shown (100 nM).

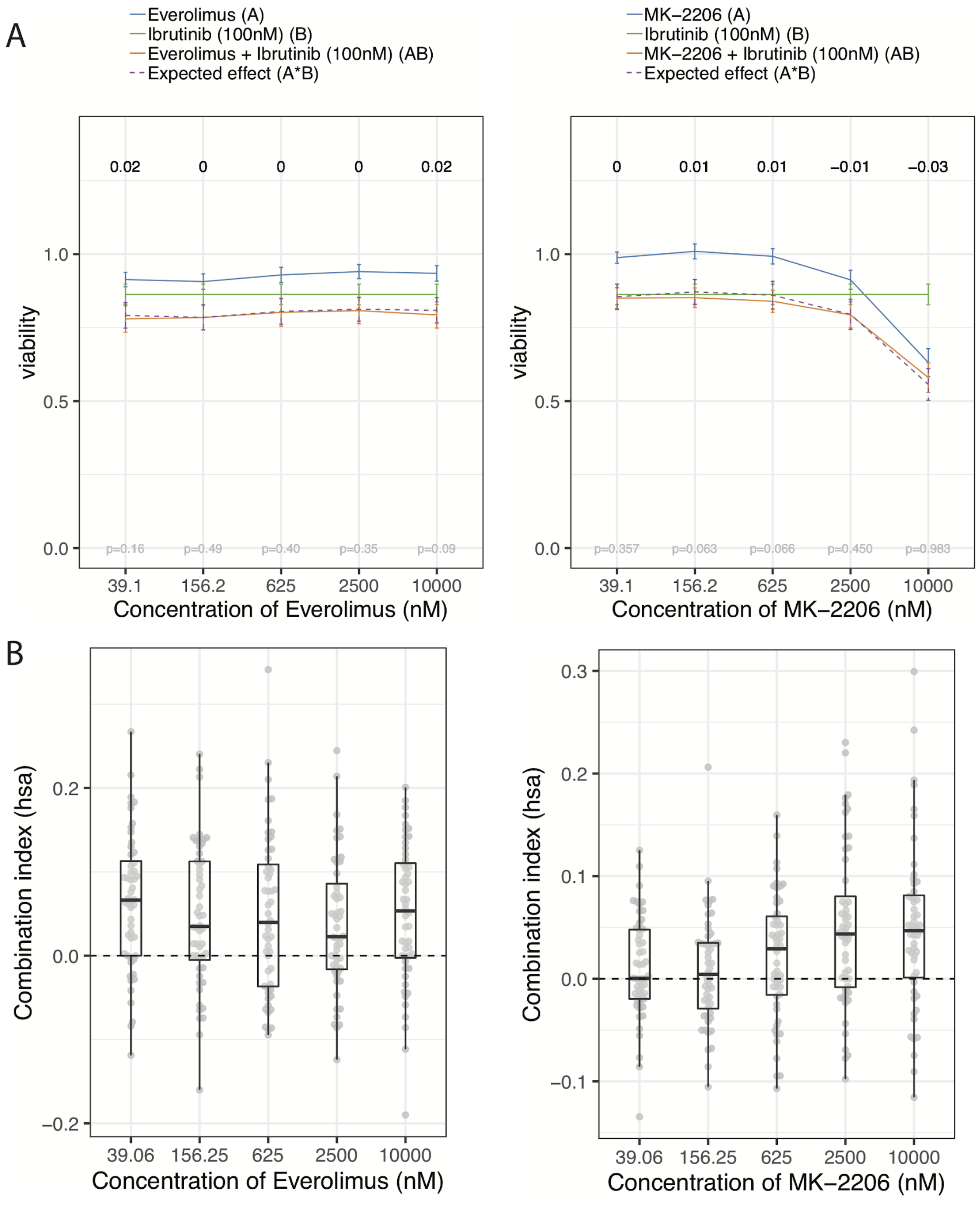

Suppl. Fig. 9: Ibrutinib in combination with distant downstream kinases

Combination activity for everolimus and MK-2206 in combination with ibrutinib 100 nM as shown by **A)** Curves representing mean viability values across 52 patient samples in response to single agents (everolimus/MK-2206/ibrutinib), to combinations with ibrutinib 100 nM and expected combination effects according to the independent effect model (A*B, **Methods**). Numbers on top indicate the average difference between the measured and expected effect at individual concentrations, p-values based on a one-sided paired *t*-test on the differences at each concentration are indicated at the bottom.

B) Box plots showing the combination index (CI) across patient samples as determined by the Highest Single Agent Approach, dots represent the Highest Single Agent (HSA) index for individual samples. No synergistic effect was observed as shown by values in the range of 0.

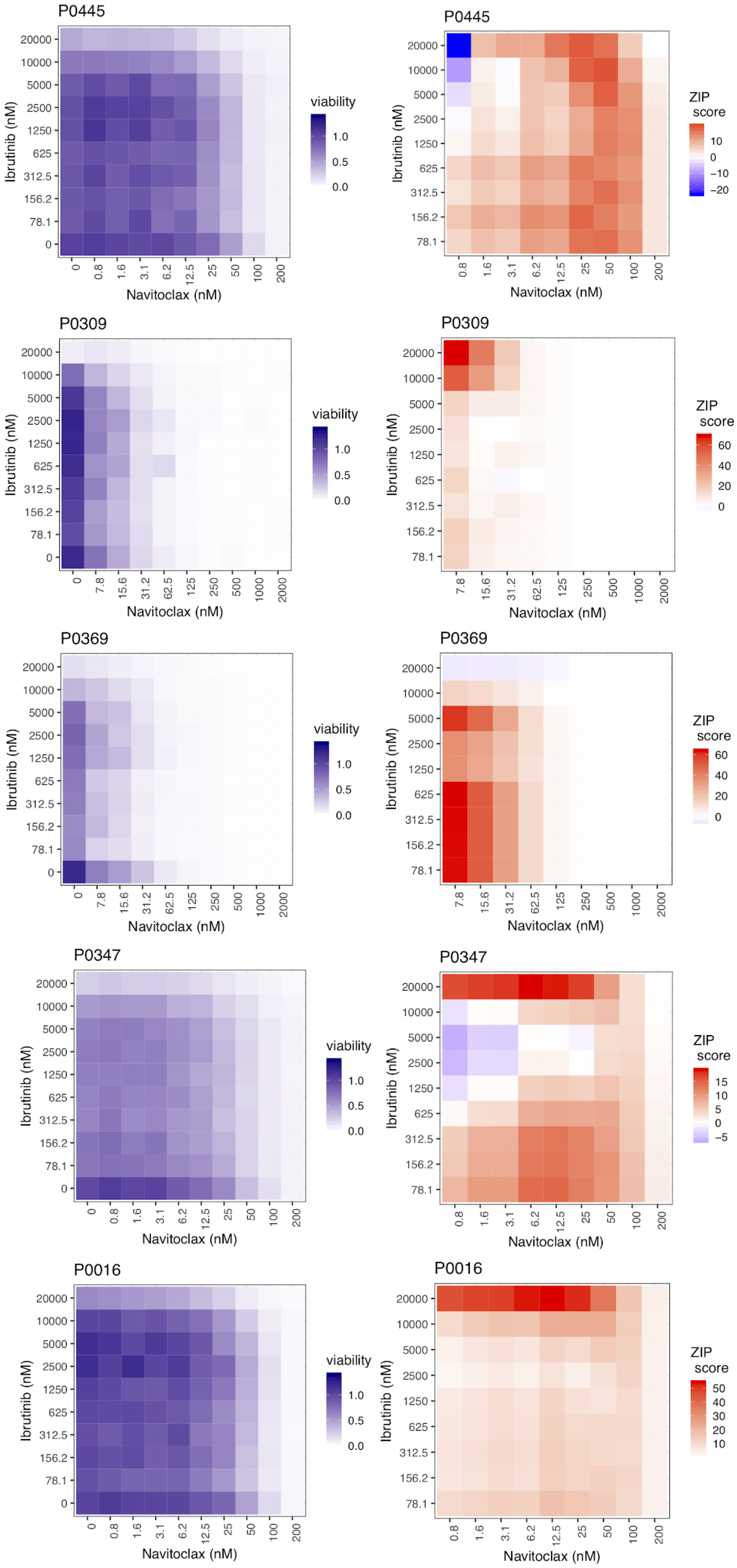

P0445
Ibrutinib (nM)
20000
10000
5000
2500
1250
625
312.5
156.2
78.1
0
Navitoclax (nM)
0
0.8
1.6
3.1
6.2
12.5
25
50
100
200
viability
1.0
0.5
0.0
P0445
ZIP score
10
0
−10
−20
P0309
Navitoclax (nM)
0
7.8
15.6
31.2
62.5
125
250
500
1000
2000
viability
P0309
ZIP score
60
40
20
0
P0369
viability
P0369
ZIP score
P0347
viability
P0347
ZIP score
15
10
5
0
−5
P0016
viability
P0016
ZIP score
50
40
30
20
10

Suppl. Fig. 10: Ibrutinib in combination with navitoclax

Left: Viability values for 5 primary CLL samples in a 10 × 10 matrix for varying concentrations of navitoclax (x-axis) and ibrutinib (y-axis). Concentrations of navitoclax range from 2 µM to 7.8 nM (2 samples) and 200 nM to 0.78 nM (3 samples) and concentrations of ibrutinib range from 78 nM to 20 µM (all samples).

Right: Combination activity for navitoclax and ibrutinib in the 5 primary CLL samples as visualized by 10 × 10 matrix block experiment and assessed using the ZIP score (**Methods**). The heatmap indicates the combination index for each concentration pair with red denoting synergistic effects (>0) and blue lack of synergy (<0).

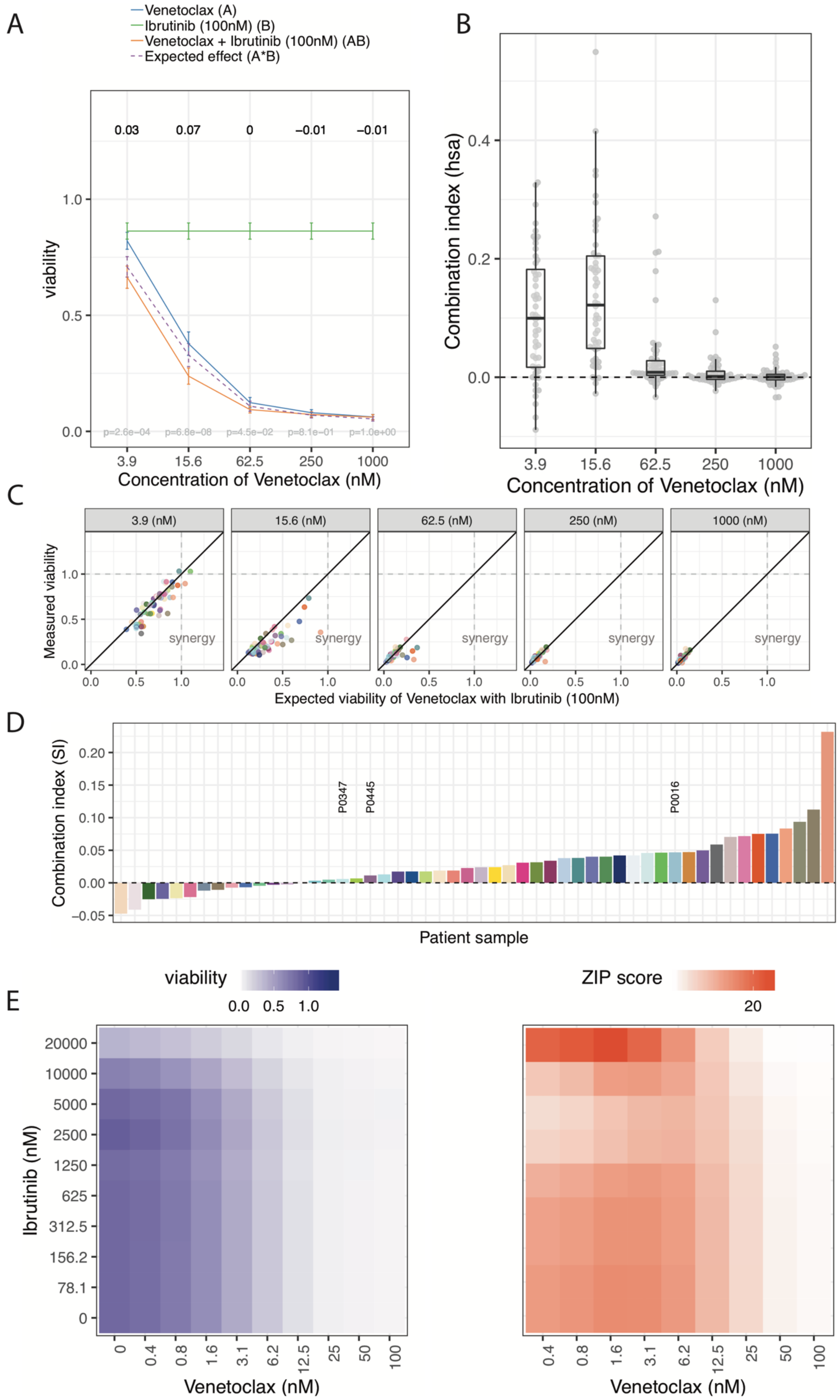
A
Venetoclax (A)
Ibrutinib (100nM) (B)
Venetoclax + Ibrutinib (100nM) (AB)
Expected effect (A*B)
0.03
0.07
0
−0.01
−0.01
viability
Concentration of Venetoclax (nM)
B
Combination index (hsa)
Concentration of Venetoclax (nM)
C
3.9 (nM)
15.6 (nM)
62.5 (nM)
250 (nM)
1000 (nM)
Measured viability
synergy
Expected viability of Venetoclax with Ibrutinib (100nM)
D
Combination index (SI)
P0347
P0445
P0016
Patient sample
E
viability
ZIP score
Ibrutinib (nM)
Venetoclax (nM)
Venetoclax (nM)

Suppl. Fig. 11: Effect of venetoclax and the combination with ibrutinib

A) Drug response curves representing mean viability values across 52 samples in response to venetoclax and ibrutinib 100 nM as single-agents (A, B), in combination (AB) and as calculated by the independent effect model (A*B, **Methods**). Numbers on top indicate the average difference between the measured and expected effect at individual concentrations, p-values based on a one-sided paired *t*-test on the differences at each concentration are indicated at the bottom. At lower concentrations [16 nM - 3.9 nM], the observed effect of the combination (AB) was stronger than the effect predicted by the independent effect model (A*B).

B) Boxplots showing the combination indices (CI) according to the highest single agent model (HSA) at individual concentrations of venetoclax. Synergistic effects are observed from 16 nM - 3.9 nM.

C) Scatterplot showing the relationship between measured combination effect AB (viability values on the y-axis) and expected combination effect A*B based on the independent effect model (viability values on the x-axis) per patient sample (colored dots). Panels correspond to individual concentrations of afatinib [1 µM – 3.9 nM]. The number of synergistic patient samples (SI > 0) at a certain concentration is given by the number of data points below the diagonal (viability values AB < viability values A*B). The synergistic effect is mainly driven by low concentrations 15.6 nM – 3.9 nM.

D) Barplots showing the synergy indices (SI) as determined by the independent effect model per patient sample (*n*=52). Values above 0 indicate synergy. Labelled patient samples were included in the 10 × 10 validation screen shown in panel **E)**.

E) Combination activity for venetoclax and ibrutinib as visualized by 10 × 10 matrix block experiment with ibrutinib concentrations ranging from 7.8 nM to 2 µM and venetoclax from 0.39 nM to 100 nM. The heatmap indicates the viability values (left) and the ZIP scores (right) at each pair of concentrations. ZIP score values shown in the heatmap were smoothed, using the mean ZIP score across 3 primary CLL samples in a running 3 × 3 concentration window. A positive value (red) denotes synergistic effects and a negative value lack of synergy (blue).

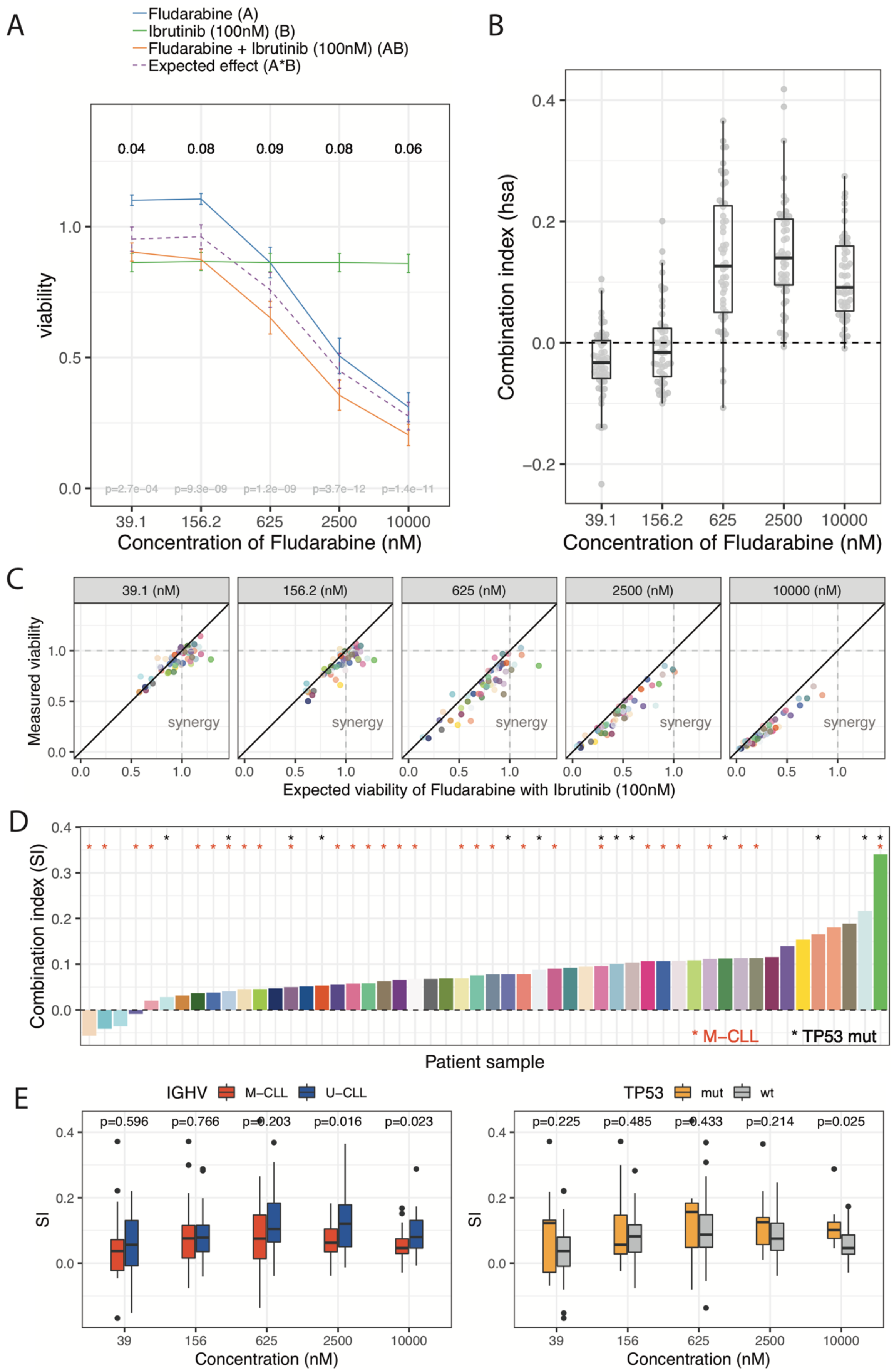

Suppl. Fig. 12: Cooperative effect of fludarabine in combination with ibrutinib

Combination responses for fludarabine and ibrutinib as judged by:

A) Drug response curves representing mean viability values across 52 samples in response to fludarabine and ibrutinib 100 nM as single-agents (A, B), in combination (AB) and as calculated by the independent effect model (A*B, **Methods**). Numbers on top indicate the average difference between the measured and expected effect at individual concentrations, p-values based on a one-sided paired *t*-test on the differences at each concentration are indicated at the bottom. Strongest synergistic combination effects were observed at higher concentrations 10 µM - 625 nM.
B) Boxplots showing the combination indices (CI) according to the highest single agent model (HSA) at individual concentrations of fludarabine. Synergistic effects are observed for 10 µM - 625 nM.
C) Scatterplot showing the relationship between measured combination effect AB (viability values on the y-axis) and expected combination effect A*B based on the independent effect model (viability values on the x-axis) per patient sample (colored dots, *n*=52). Panels correspond to individual concentrations of fludarabine [10 µM – 39 nM]. The number of synergistic patient samples (SI > 0) at a certain concentration is given by the number of data points below the diagonal (viability values AB < viability values A*B). Synergy is driven by concentrations 10 µM - 625 nM.
D) Barplots showing the synergy indices (SI) as determined by the independent effect model per patient sample (*n*=52). Values above 0 indicate synergy (*n*=48). Bars are annotated by IGHV status (red) and presence of TP53 mutations (black).
E) Box plots showing synergy indices of the samples grouped according to TP53 and IGHV status for individual concentrations of fludarabine. U-CLL samples show consistently higher synergy indices across concentrations, TP53 status did not show a clear influence on synergy.

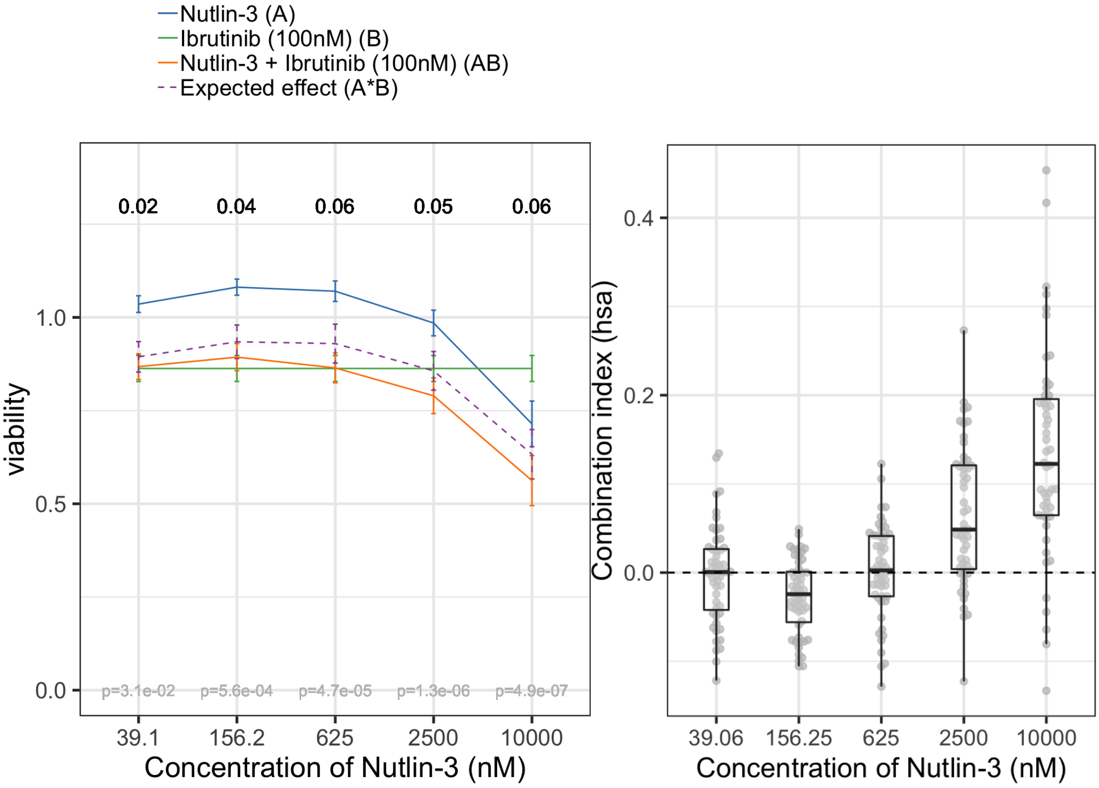

Suppl. Fig. 13: Cooperative effect of nutlin-3 in combination with ibrutinib

A) Drug response curves representing mean viability values across 52 samples in response to nutlin-3 and ibrutinib 100 nM as single-agents (A, B), in combination (AB) and as calculated by the independent effect model (A*B, **Methods**). Numbers on top indicate the average difference between the measured and expected effect at individual concentrations, p-values based on a one-sided paired *t*-test on the differences at each concentration are indicated at the bottom. Synergistic combination effects were observed at higher concentrations [10 µM – 2.5 µM].

B) Boxplots showing the combination indices (CI) according to the highest single agent model (HSA) at individual concentrations of nutlin-3. Synergistic effects are observed at higher concentrations [10 µM – 2.5 µM].

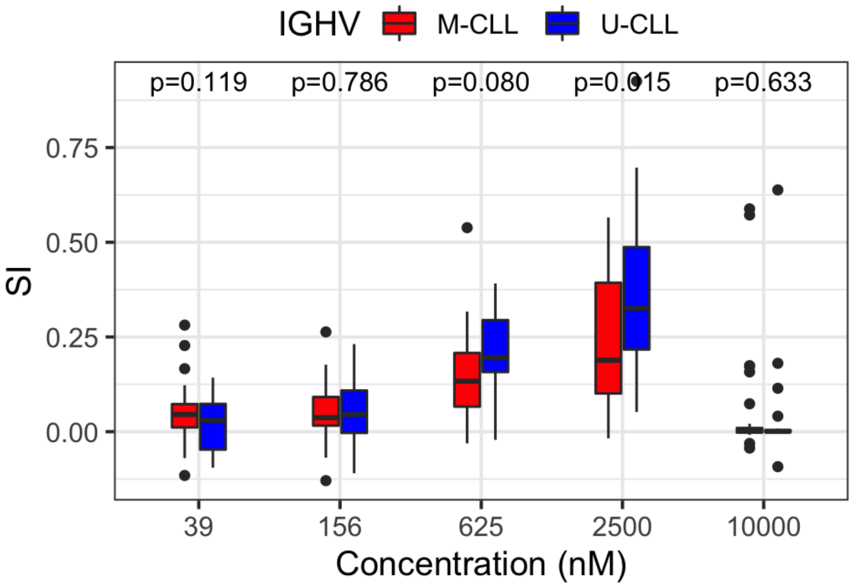

Suppl. Fig. 14: Influence of IGHV status on synergy of afatinib and ibrutinib
Boxplots show the synergy indices at different concentrations of afatinib for samples stratified by IGHV status. P-values indicated on top are based on a *t*-test between the groups.

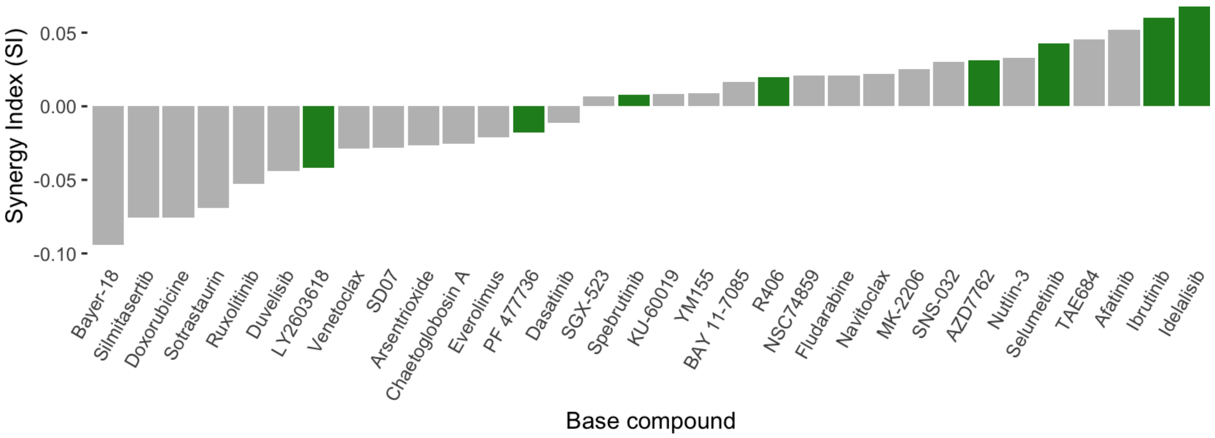

Suppl. Fig. 15: Cooperative effects with afatinib

Synergy indices (SI) for afatinib and the 32 drug library as calculated by the independent effect model (**Methods**) across *n*=30 samples are shown. Values above 0 indicate synergy (*n*=18). Combinations with afatinib resulted in synergistic effects with several inhibitors of the BCR, of MEK and of CHK (indicated in green).

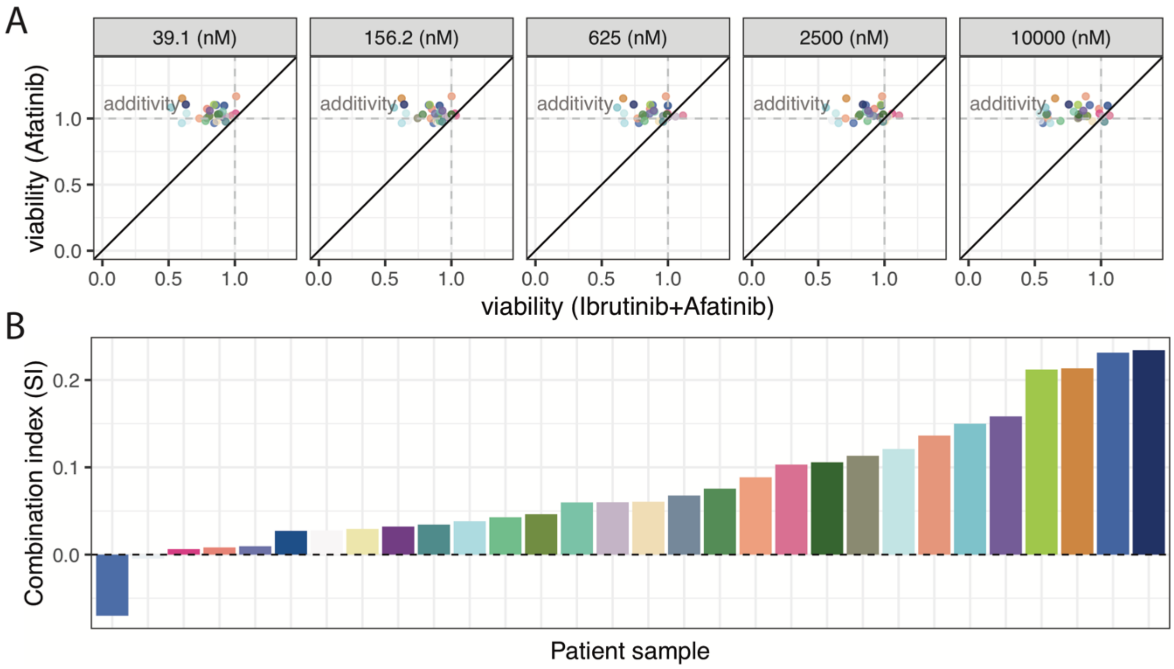

Suppl. Fig. 16: Abolition of prosurvival effects induced by afatinib 100 nM

A) Viability values in response to afatinib 100 nM (y-axis) versus the combination with library drug ibrutinib (x-axis) across concentrations [10 µM – 39 nM] are shown. Prosurvival effects induced by afatinib can be antagonized by ibrutinib across concentrations [10 µM – 39 nM].

B) Barplots showing the synergy indices (SI) for combination of afatinib with ibrutinib as determined by the independent effect model per patient sample (*n*=30). Values above 0 indicate synergy (*n*=28) with a median synergy index of 0.06.

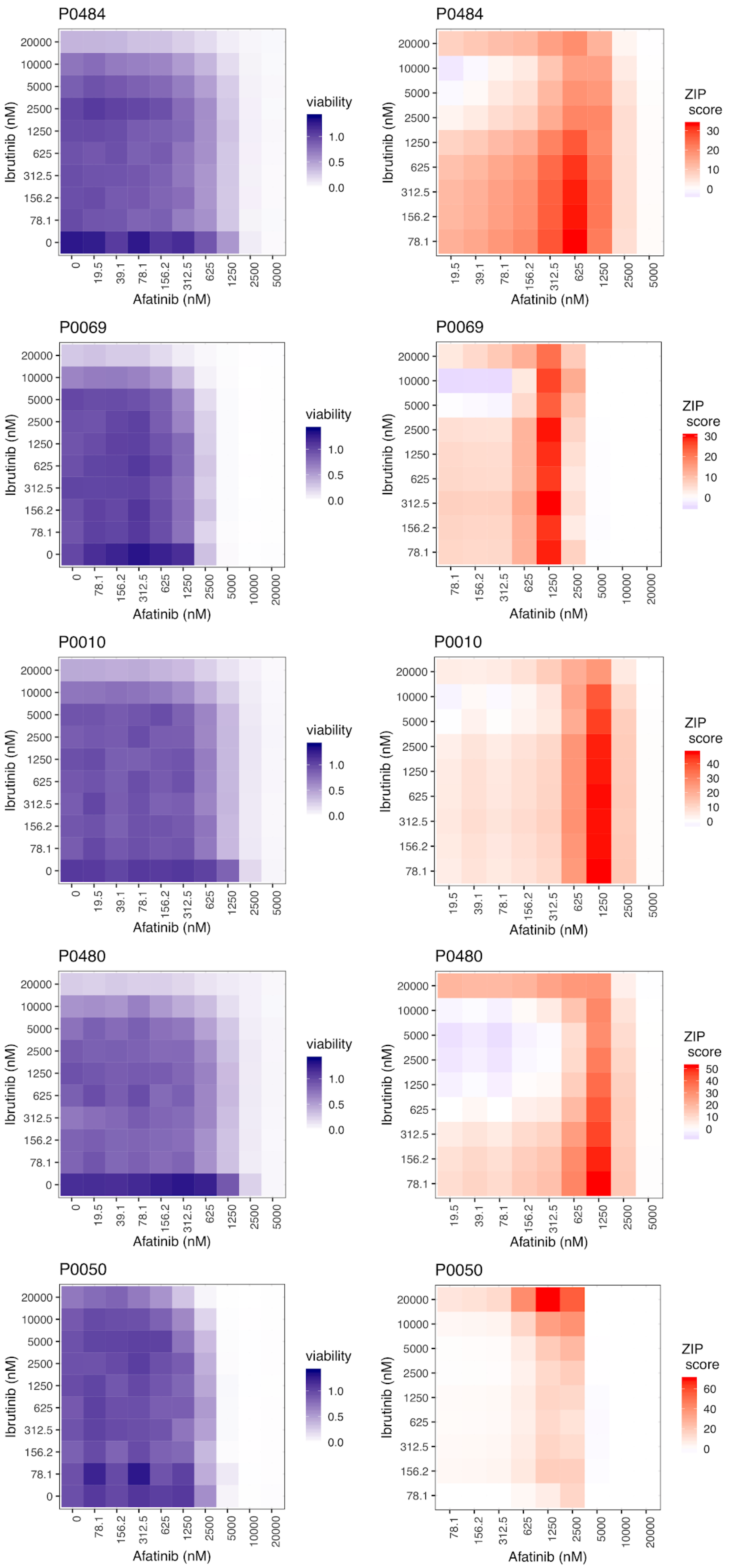
P0484
Ibrutinib (nM)
Afatinib (nM)
viability
1.0
0.5
0.0
P0484
Ibrutinib (nM)
Afatinib (nM)
ZIP score
30
20
10
0
P0069
Ibrutinib (nM)
Afatinib (nM)
viability
P0069
Ibrutinib (nM)
Afatinib (nM)
ZIP score
P0010
Ibrutinib (nM)
Afatinib (nM)
viability
P0010
Ibrutinib (nM)
Afatinib (nM)
ZIP score
40
30
20
10
0
P0480
Ibrutinib (nM)
Afatinib (nM)
viability
P0480
Ibrutinib (nM)
Afatinib (nM)
ZIP score
50
40
30
20
10
0
P0050
Ibrutinib (nM)
Afatinib (nM)
viability
P0050
Ibrutinib (nM)
Afatinib (nM)
ZIP score
60
40
20
0

Suppl. Fig. 17: Ibrutinib in combination with afatinib

For 5 primary CLL samples we show

Left: Viability values in the 10 × 10 matrix for varying doses of afatinb (x-axis) and ibrutinib (y-axis). **Right:** Combination activity for afatinb and ibrutinib as visualized by 10 × 10 matrix block experiment and assessed using the ZIP score (**Methods**). The heatmap indicates the ZIP score for each concentration pair with red denoting synergistic effects (>0) and blue lack of synergy (<0).

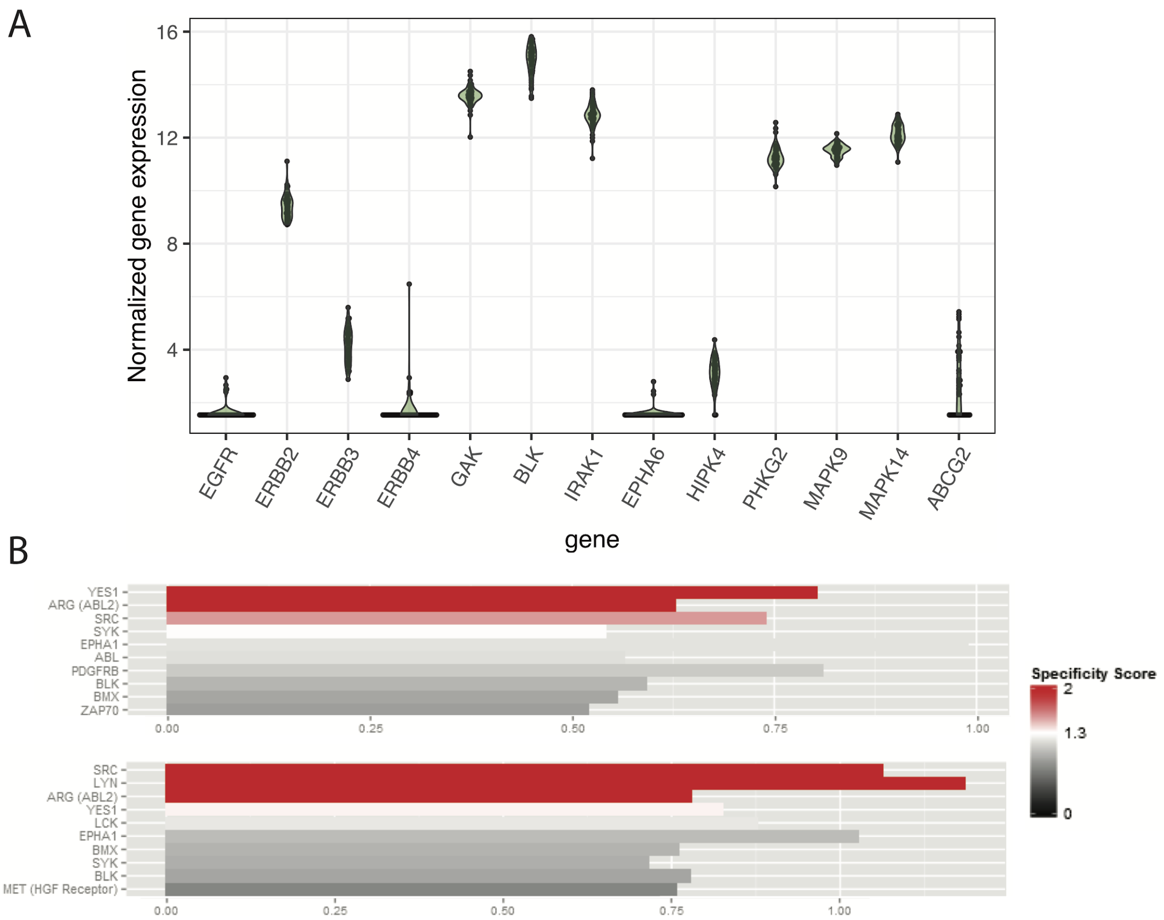

Suppl. Fig. 18: Pamgene chip assay identifies direct and indirect targets of afatinib

A) Violin plots of normalized gene expression values for potential afatinib targets. Points represent expression values of the *n*=52 samples. Largest expression values were observed for BLK, which is upstream of BTK.

B) Three primary CLL samples sensitive to afatinib were treated with 1.67 µM afatinib after (top) or before (bottom) cell lysis and resulting cellular lysates were analyzed for kinase activity on the PamStation® 12 system with the PamChip® Tyrosine Kinase Array Chips (**Methods**). Top ten results of predicted upstream kinases are shown.

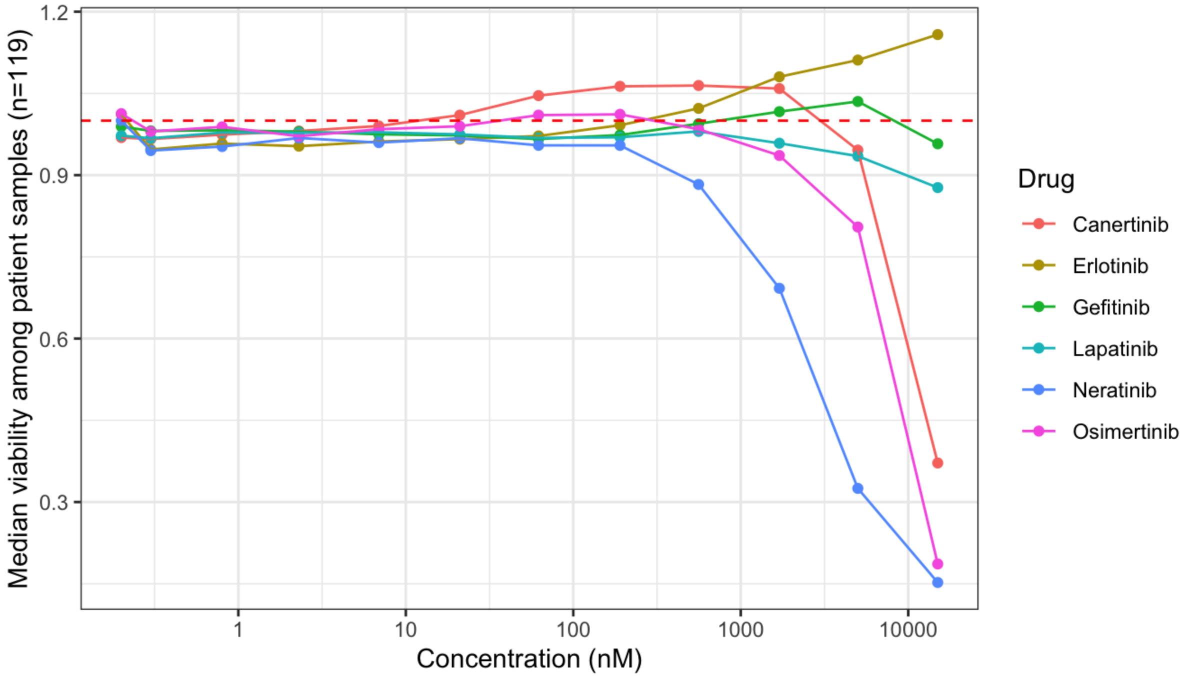

Suppl. Fig. 19: Response curves of EGFR inhibitors

Drug-response curves of six EGFR inhibitors. On the x-axis the concentration of the compound is shown, the y-axis shows the median viability value across n=119 patient samples. Each line and color represents one EGFR inhibitor.

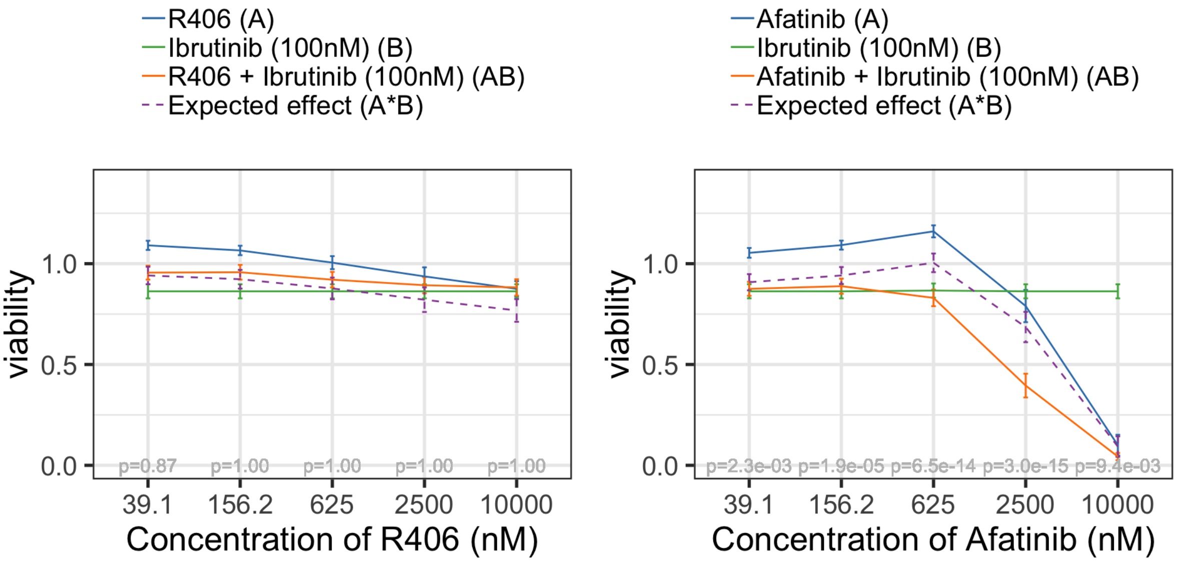

Suppl. Fig. 20: Comparison of drug response curves for R406 and afatinib in combination with Ibrutinib

Effects of single-agents (A, B), measured combination effect (AB) and expected combination effect according to the independent effect model (A*B) for Ibrutinib with R406 (left) or afatinib (right). The curves represent the mean viability values with error bars denoting two standard errors, p-values based on a one-sided paired *t*-test on the differences at each concentration are indicated at the bottom.

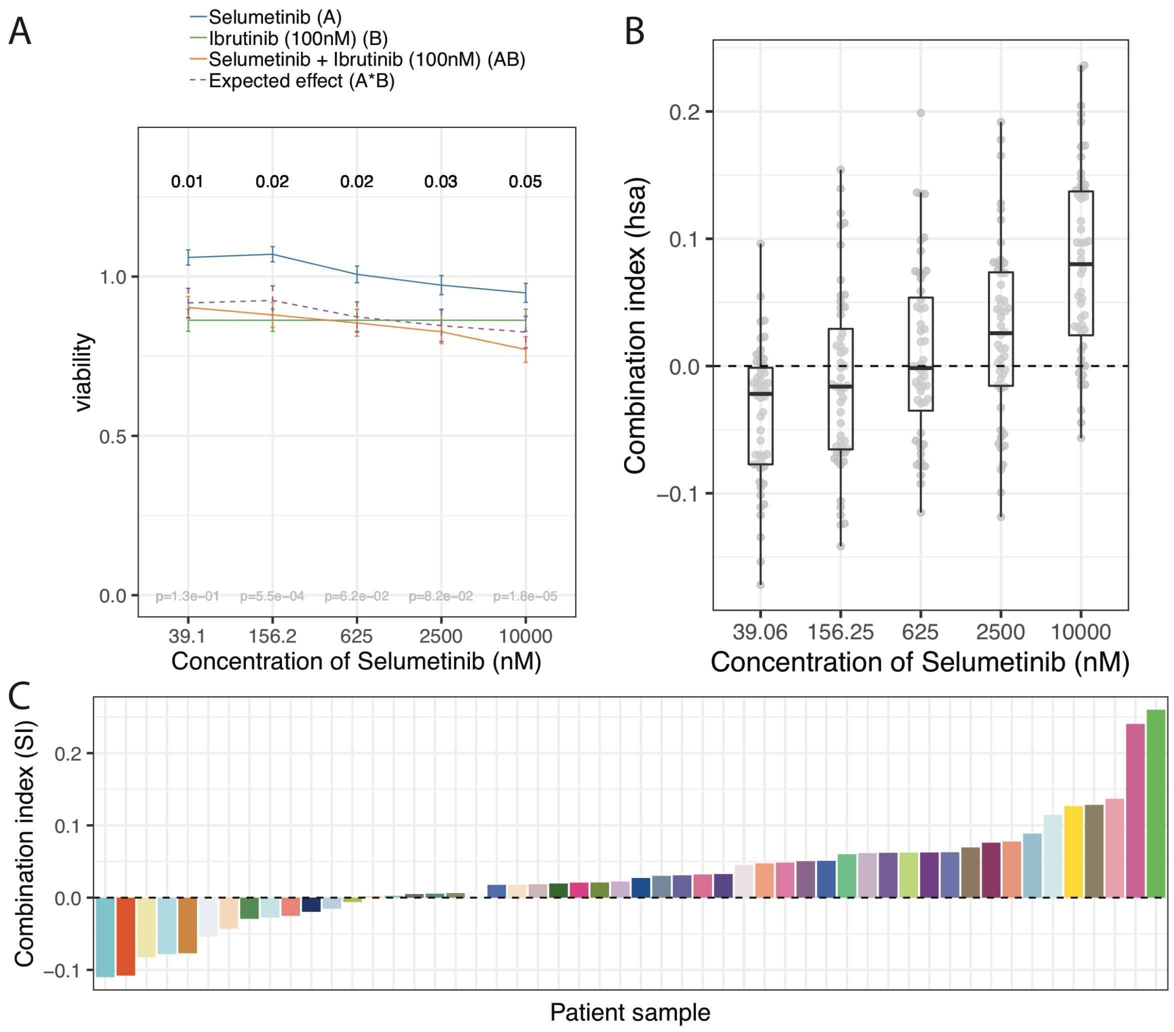

Suppl. Fig. 21: Ibrutinib and selumetinib in combination

A) Drug response curves representing mean viability values across 52 samples in response to selumetinib and ibrutinib 100 nM as single-agents (A, B), in combination (AB) and as calculated by the independent effect model (A*B, **Methods**). Numbers on top indicate the average difference between the measured and expected effect at individual concentrations, p-values based on a one-sided paired *t*-test on the differences at each concentration are indicated at the bottom. Synergistic combination effects were observed at higher concentrations [10 µM – 2.5 µM].

B) Boxplots showing the combination indices (CI) according to the highest single agent model (HSA) at individual concentrations of selumetinib. Synergistic effects are observed for [10 µM – 2.5 µM].

C) Barplots showing the synergy indices (SI) as determined by the independent effect model per patient sample (*n*=52). Values above 0 indicate synergism (*n*=37).

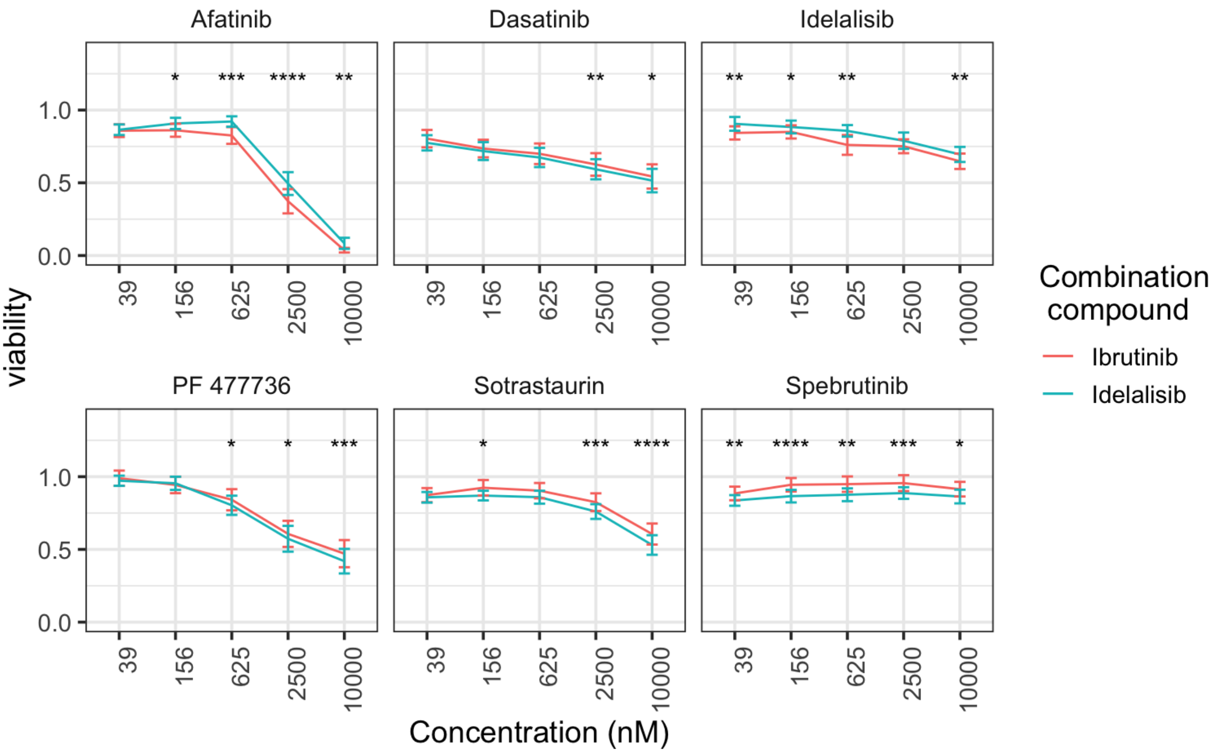

Suppl. Fig. 22: Ibrutinib and idelalisib in combination

Drug response curves showing the mean viability in response to the drug combination for library drugs that showed a different response profile for combinations with ibrutinib (red) compared to idelalisib (blue). Error bars denote two standard errors. P-values at individual concentrations were assessed using Student's *t*-test with paired samples. (*: $p <= 0.05$, **: $p <= 0.01$, ***: $p <= 0.001$,****: $p <= 0.0001$)

Suppl. Table 1: Drugs and commercial suppliers

Compound	Main targets	Distributor
Navitoclax	Bcl-2	Selleck Chemicals
Ibrutinib	Btk	Selleck Chemicals
Idelalisib	PI3Kδ	Selleck Chemicals
SNS-032	CDK 2,7,9	Selleck Chemicals
Fludarabine	purine analogue	Selleck Chemicals
Nutlin-3	MDM2	Selleck Chemicals
Selumetinib	MEK1/2	Selleck Chemicals
Afatinib	EGFR/ERBB2	Selleck Chemicals
AZD7762	CHK1/2	Selleck Chemicals
NSC 74859 (S3I-201)	STAT	Selleck Chemicals
TAE684 (NVP-TAE684)	ALK	Selleck Chemicals
MK-2206 dihydrochloride	AKT/PKB	Selleck Chemicals
R406	Syk	Selleck Chemicals
YM155	Survivin	Selleck Chemicals
BAY 11-7085	NF-kB	Enzo Life Sciences
SGX-523	c-MET	Selleck Chemicals
KU-60019	ATM	Selleck Chemicals
Chaetoglobosin A	fungal toxin	Enzo Life Sciences
Dasatinib	Src/Abl	Selleck Chemicals
Everolimus	mTOR	Selleck Chemicals
Arsentrioxide	chemotherapeutic agent	Sigma-Aldrich
LY2603618 (IC-83)	CHK1	Selleck Chemicals
PF 477736	CHK1/2, VEGFR2, Yes, Fms	Selleck Chemicals

Sprebrutinib	Btk	Selleck Chemicals
Venetoclax	Bcl-2	Selleck Chemicals
Duvelisib	PI3K δ, PI3K γ	Selleck Chemicals
Ruxolitinib	Jak1/2/3	Selleck Chemicals
SD07	ROS	Academic cooperation
Doxorubicine	DNA interkalation, Topoisomerase II	Sigma-Aldrich
Bayer-18	Tyk2	Academic cooperation
Silmitasertib	CK2	Selleck Chemicals
Sotrastaurin (AEB071)	pan PKC	Selleck Chemicals
Pomalidomide	immune modulatory	Academic cooperation
Encorafenib	BRAF	Academic cooperation
